# A ROS-responsive platelet extracellular vesicle biomimetic nanosystem regulates CD36/Fyn-CXCL-NETosis pathway-mediated immune-inflammatory amplification in the treatment of transfusion-related acute lung injury

**DOI:** 10.1016/j.redox.2026.104375

**Published:** 2026-09-06

**Authors:** Jiani Gao, Ke Zhou, Yijiu Ren, Fengjing Yang, Minjue Shan, Yubing Fang, Changbo Sun, Weili Han, Chang Chen, Xuefei Hu

**Affiliations:** a Department of Thoracic Surgery, Shanghai Pulmonary Hospital, Tongji University School of Medicine, Shanghai, 200433, China; b Department of Thoracic Surgery, Union Hospital, Tongji Medical College, Huazhong University of Science and Technology, Wuhan, China; c Clinical Center for Thoracic Surgery Research, Tongji University, Shanghai, China; d Tongji University School of Medicine, Shanghai, China; e Department of Lung Transplantation and Thoracic Surgery, The First Affiliated Hospital, Zhejiang University School of Medicine, Hangzhou, 310003, China

**Keywords:** Transfusion-related acute lung injury, CD36/Fyn signaling axis, Biomimetic nanosystem, Neutrophil extracellular trap-osis, Single-cell RNA sequencing, Immune-inflammatory amplification

## Abstract

Transfusion-related acute lung injury (TRALI) remains a life-threatening complication of blood transfusion, yet the macrophage-neutrophil-NETosis inflammatory amplification mechanism and effective interventions are still incompletely defined. Here, we examined a CD36/Fyn-CXCL-related axis in TRALI and evaluated a ROS-responsive platelet extracellular vesicle biomimetic nanosystem (PEV@SP@EG) as a targeted delivery strategy. In a murine TRALI model, exploratory single-cell transcriptomic analysis showed immune-cell remodeling and linked CD36 expression to M1-like macrophage features. CD36 knockdown or pharmacological inhibition of Fyn/NF-kappaB reduced M1 polarization markers, CXCL production, and ROS generation. Conditioned medium from CD36-overexpressing BMDMs promoted neutrophil migration and NETosis, whereas CXCL-CXCR2 blockade or CD36/Fyn inhibition weakened these responses. PEV@SP@EG showed ROS-triggered drug release and preferential lung enrichment. *In vivo*, PEV@SP@EG attenuated CD36/Fyn-NF-kappaB-CXCL-related inflammatory signaling, NET formation, and oxidative stress in TRALI lung tissue, with concomitant improvement in lung architecture and gas exchange. These findings support a role for macrophage CD36/Fyn signaling in TRALI-associated macrophage-neutrophil inflammatory crosstalk and suggest that PEV@SP@EG may provide a targeted nanotherapeutic delivery platform for TRALI intervention.

## Graphical abstract

Mechanistic Schematic Showing How PEV@SP@EG Regulates CD36/Fyn-NF-κB-CXCL-Related Inflammatory Signaling to Alleviate Macrophage Pro-Inflammatory Polarization and Neutrophil NETosis.

**Figure:**
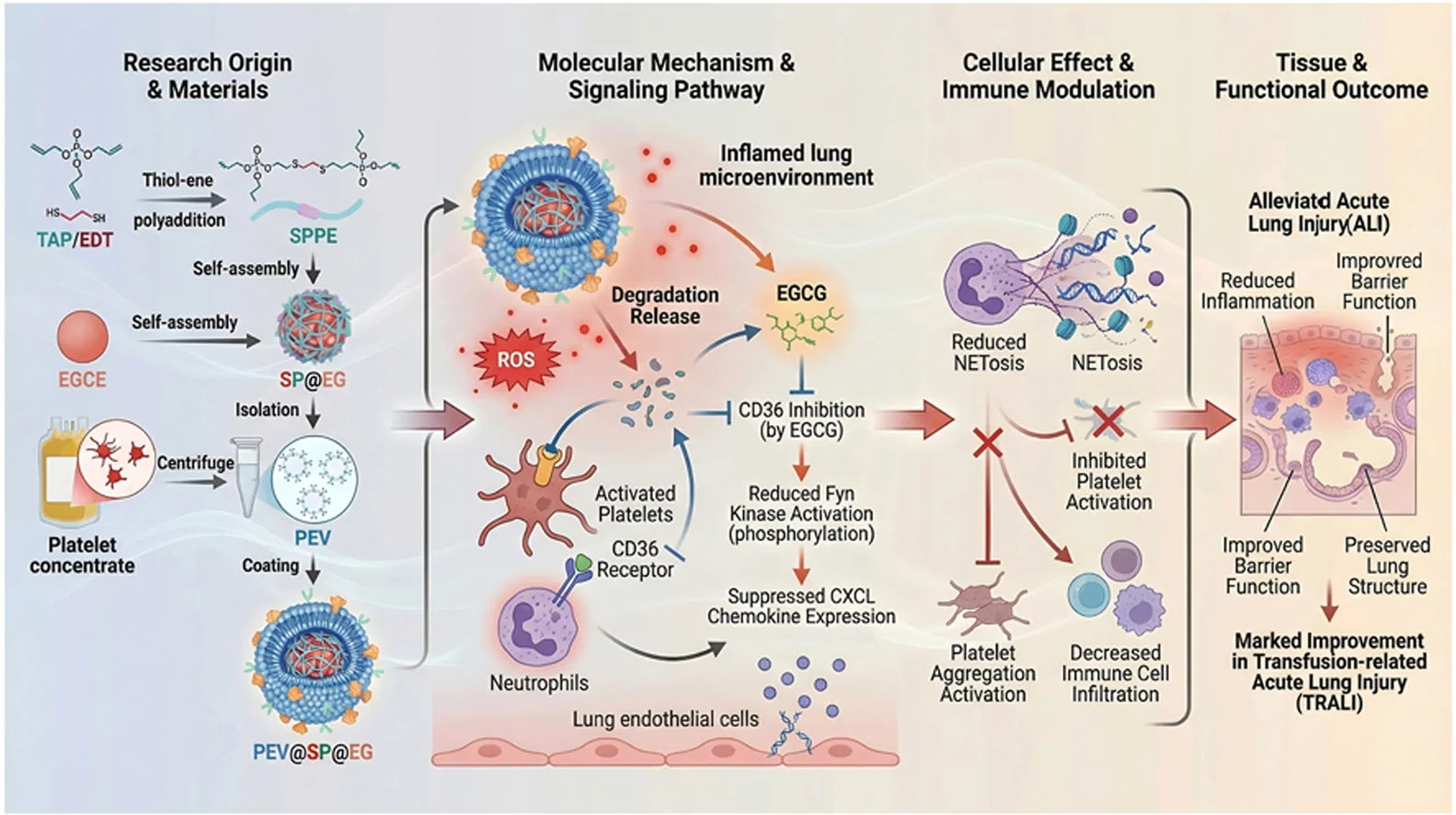

## 1 Introduction

Transfusion-related acute lung injury (TRALI) is currently one of the most severe complications during the peri-transfusion period, characterized by acute onset, rapid progression, and high mortality [1]. Its typical clinical manifestations include non-cardiogenic pulmonary edema, hypoxemia, and bilateral pulmonary infiltrates [2]. In severe cases, TRALI can lead to respiratory failure, posing a serious threat to patient survival [3]. Despite continuous improvements in modern transfusion management, TRALI still occurs, and its incidence across different blood components remains non-negligible [4]. Due to its diagnosis being based on exclusion criteria and the current lack of specific biomarkers or effective targeted therapies, clinical management primarily relies on supportive care, which greatly limits treatment success rates and patient quality of life [1,5]. Therefore, there is an urgent need to identify key molecular targets at the mechanistic level to inform the development of precision therapeutic strategies.

An increasing number of studies have indicated that a robust immune-inflammatory response lies at the core of TRALI pathogenesis, particularly the inflammatory amplification cascade involving macrophages, neutrophils, and neutrophil extracellular trap-Osis (NETosis) formation [6]. M1-type macrophages are activated during TRALI and promote neutrophil-mediated pulmonary inflammatory responses through the release of pro-inflammatory mediators [7,8]. While neutrophil extracellular traps (NETs) released by neutrophils play a crucial role in antimicrobial defense, their excessive formation compromises the alveolar-capillary barrier, triggering extensive pulmonary tissue injury and impaired oxygenation [9,10]. This inflammatory positive feedback loop is considered a key driver of TRALI's rapid clinical deterioration [11]. However, current understanding of the upstream regulatory signals involved in this process remains limited. Existing interventions primarily target downstream inflammatory mediators, lacking both specificity and mechanistic precision, highlighting the urgent need to identify novel regulatory hub molecules [10].

CD36 is a multifunctional transmembrane receptor widely expressed on the surface of macrophages, platelets, and endothelial cells, and is involved in various physiological processes, including lipid metabolism, immune recognition, and inflammatory signal transduction [12,13]. Previous studies have shown that CD36 can activate the downstream tyrosine kinase Fyn, which in turn triggers the NF-κB pathway, thereby promoting the expression of inflammatory and chemotactic factors, inducing M1 polarization, and enhancing oxidative stress responses [14]. Notably, in diseases such as atherosclerosis, the CD36/Fyn axis has been identified as a central regulator of inflammation [15]. However, whether CD36 exerts a similar role in TRALI—a lung injury model dominated by immune amplification—remains unconfirmed. In recent years, novel nanomedicine-based intervention strategies have shown promising potential in the treatment of inflammatory diseases [16,17]. Platelet-derived extracellular vesicles (PEVs), due to their intrinsic immune regulatory functions, pulmonary homing ability, and excellent biocompatibility, have emerged as promising drug-delivery platforms. Accordingly, we established cellular and murine TRALI models to define the role of CD36/Fyn-NF-kappaB-CXCL signaling in macrophage polarization, neutrophil recruitment, and NETosis, and to evaluate whether ROS-responsive PEV@SP@EG could provide targeted anti-inflammatory protection in this setting. The overall experimental design and workflow are summarized in the Flowchart.

**Figure:**
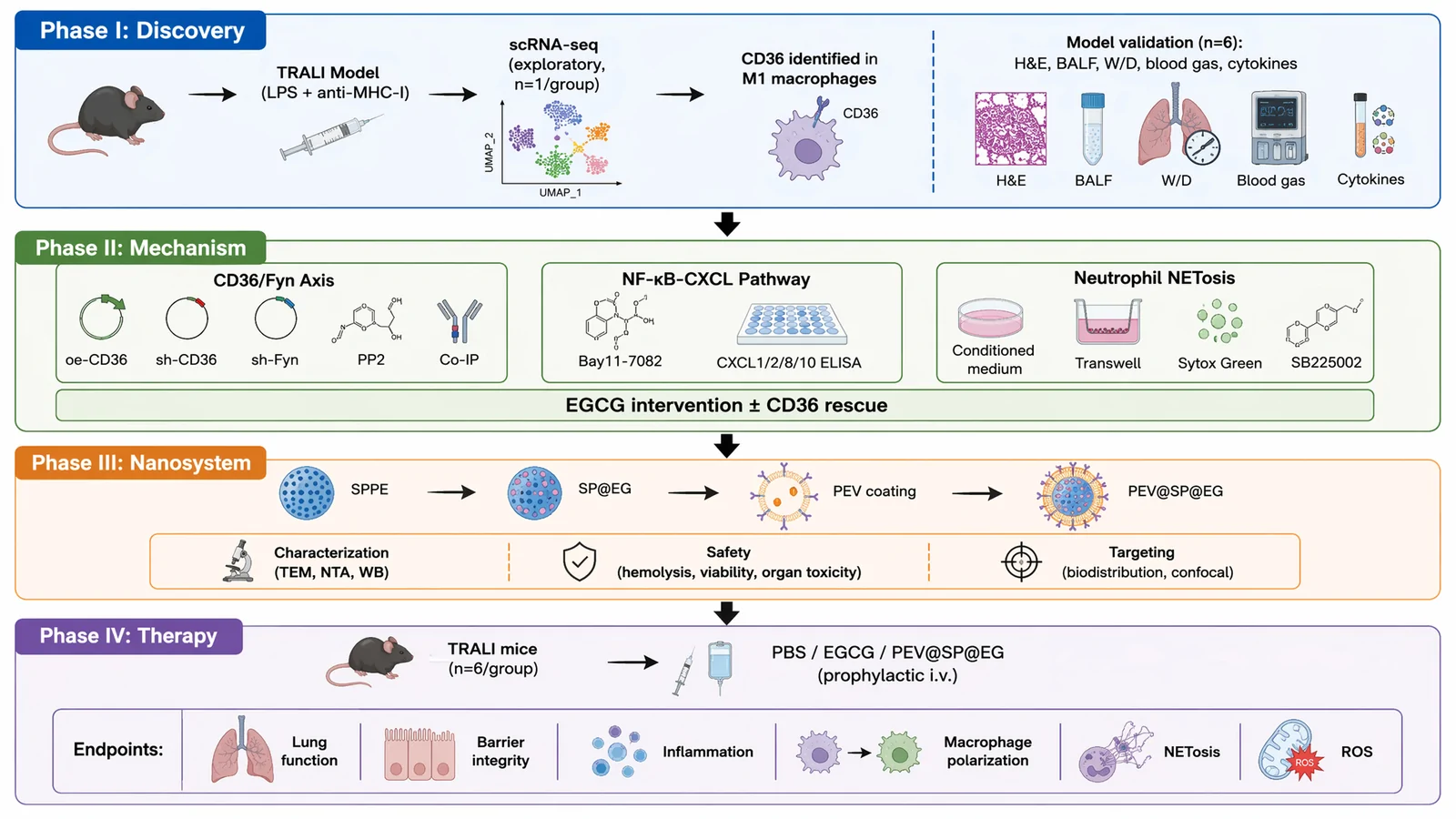
Flowchart. Overall experimental workflow covering discovery, mechanistic validation, nanosystem construction, and therapeutic evaluation in the TRALI model.

## 2 Materials and methods

### Ethical statement

This study strictly adhered to the relevant ethical guidelines and regulations for animal experimentation. All procedures were approved by the Institutional Animal Care and Use Committee (IACUC) of our institution (Approval Number: 2022-011562). All animals were housed and cared for under humane conditions, and experiments were conducted with efforts to minimize pain and distress. At the conclusion of the experiments, all mice were humanely euthanized under sodium pentobarbital anesthesia.

### 2.1 Synthesis of SPPE polymer

To obtain a polymer with ROS-responsive properties, SPPE was synthesized via a thiol-ene addition reaction. Triallyl phosphate (TAP; CAS No. 2417-54-5, #2417545, Sigma-Aldrich, USA) and 1,2-ethanedithiol (EDT; E114603, Aladdin, China) were dissolved in anhydrous dimethylformamide (DMF; D819015, Macklin, China) at a 1:1 M ratio. A photoinitiator, Irgacure 2959 (2-Hydroxy-4′-(2-hydroxyethoxy)-2-methylpropiophenone; 410896, Sigma-Aldrich, USA), was added at a concentration of 0.1 wt%. The mixture was irradiated with 365 nm ultraviolet light under nitrogen protection using a UV curing system (10 mW/cm^2^) for 4 h. After completion of the reaction, the solvent was removed using a rotary evaporator (RE-52AA, Yarong, China) at 40 °C. The residual product was subsequently freeze-dried using a lyophilizer (FreeZone 2.5, Labconco, USA) to obtain a pale-yellow solid SPPE polymer.

### 2.2 Preparation of Epigallocatechin Gallate-loaded sulfur-containing Poly(Phosphate Ester) nanoparticles (SP@EG)

EGCG-loaded nanoparticles were prepared using the nanoprecipitation method. EGCG (≥95%, HY-N0083, MedChemExpress, USA) was dissolved in anhydrous ethanol at a concentration of 10 mg/mL and slowly added dropwise at a rate of 0.5 mL/min into deionized water containing SPPE polymer (final concentration 1 mg/mL). The mixture was stirred magnetically for 2 h to allow self-assembly into nanoparticles. The resulting suspension was centrifuged at 12,000 rpm for 20 min at 4 °C to remove unencapsulated drug. The pellet was collected and resuspended in phosphate-buffered saline (PBS) to obtain the SP@EG nanoparticles.

### 2.3 Isolation and purification of PEVs

Fresh platelet samples were obtained from healthy mouse blood. Initial low-speed centrifugation was performed at 2500 ×g for 15 min at 4 °C to remove cell debris and impurities. The supernatant was collected and centrifuged again at 10,000 ×g for 20 min at 4 °C to eliminate larger particles. The subsequent supernatant was subjected to ultracentrifugation at 100,000 ×g for 70 min at 4 °C to pellet PEVs. The resulting pellet was resuspended in sterile PBS (pH 7.4) and filtered through a 0.22 μm membrane filter (Millex-GP, SLGP033RB, Millipore, USA) to remove residual impurities, yielding purified PEVs.

### 2.4 Construction of the PEV@SP@EG nanosystem

SP@EG nanoparticles and PEVs were thoroughly mixed at a mass ratio of 1:2, followed by the preparation of biomimetic nanoparticles using a sonication-assisted membrane extrusion method. Sonication was performed in an ice bath (40 kHz, 100 W, 3 min; Scientz-IID, Scientz, China), after which the mixture was extruded 10 times through a 200 nm polycarbonate membrane (pore size: 200 nm; 610007, Whatman Nuclepore Track-Etched Membrane, UK) using a mini extruder (Avanti Mini Extruder, Avanti Polar Lipids, USA). This process yielded uniform and stable biomimetic nanoparticles, with PEVs coating the SP@EG core, referred to as PEV@SP@EG. The resulting particles were stored in the dark at 4 °C for further use.

### 2.5 Transmission Electron microscopy (TEM) observation

A 10 μL aliquot of the nanoparticle suspension was dropped onto a Formvar-carbon-coated copper grid (400 mesh; 01800-F, Ted Pella, USA) and allowed to stand at room temperature for 2 min to facilitate particle adsorption. The sample was then negatively stained with 2% phosphotungstic acid solution (pH 7.0; P4006, Sigma-Aldrich, USA) for 30 s. Excess stain was gently removed using filter paper, and the grid was air-dried. The morphology of the nanoparticles was observed using a TEM (JEM-2100, JEOL, Japan) operated at an accelerating voltage of 200 kV. Images were captured with a Gatan Orius CCD camera.

### 2.6 Nanoparticle tracking analysis (NTA)

The size distribution and particle concentration of PEVs and the PEV@SP@EG nanosystem were analyzed using a NanoSight NS300 nanoparticle tracking analyzer (Malvern Panalytical, UK). Samples were diluted 1:100 in sterile PBS and measured at a constant temperature of 25 °C. For each sample, three 60-s videos were recorded at 30-s intervals. Data were processed using NTA software (NTA 3.2 Dev Build 3.2.16) to determine the Z-average diameter, size distribution, and particle concentration. During measurement, the sample chamber temperature was automatically regulated using the built-in temperature control module to ensure accuracy and reproducibility.

### 2.7 ROS responsiveness assay

To evaluate the stability and degradation behavior of the PEV@SP@EG nanosystem under oxidative conditions, the nanoparticle solution was added to hydrogen peroxide (H_2_O_2_) solutions at varying final concentrations (0, 10, 50, 100, 500, 1000, and 2000 μM; H_2_O_2_, 30% w/w, H1009, Sigma-Aldrich, USA). The mixtures were incubated at 37 °C for 30 min, after which color and turbidity changes were visually assessed. Subsequently, a UV-visible spectrophotometer (UV-2600, Shimadzu, Japan) was used to measure transmittance at 600 nm to assess the degradation behavior of the nanoparticles under ROS exposure. Three replicate transmittance measurements were obtained for each condition.

### 2.8 Drug release assay

To investigate the EGCG release behavior of PEV@SP@EG nanoparticles under different oxidative stress conditions, 1 mL of nanoparticle solution was loaded into dialysis bags with a molecular weight cutoff (MWCO) of 8-14 kDa (Spectra/Por® Dialysis Membrane, 132655, Spectrum Labs, USA). The bags were immersed in 50 mL of PBS buffer (pH 7.4, Gibco, USA) containing different concentrations of H_2_O_2_ (0 and 100 μM, Sigma-Aldrich, H1009), and incubated in a thermostatic shaker at 37 °C and 100 rpm (HZQ-X100, Taicang Laboratory Equipment Factory, China).

At predetermined time points (0, 1, 2, 4, 8, 12, 16, 24, and 48 h), 1 mL of the external solution was withdrawn and replaced with an equal volume of fresh PBS to maintain a constant volume. The concentration of EGCG in the collected samples was determined at 275 nm using a UV-visible spectrophotometer (UV-2600, Shimadzu, Japan). EGCG concentration was calculated based on a standard calibration curve, and the cumulative release percentage was subsequently determined.

### 2.9 Establishment and grouping of the murine TRALI model

Male C57BL/6J mice (6-10 weeks old, 20-25 g; strain code 219, Beijing Vital River Laboratory Animal Technology Co., Ltd., China) were housed under specific pathogen-free (SPF) conditions for 7 days before being randomly assigned to experimental groups. A two-hit model was employed to induce TRALI: the first hit consisted of intraperitoneal injection of lipopolysaccharide (LPS, 0.1 mg/kg; Sigma-Aldrich) to induce systemic inflammation and precondition the pulmonary microenvironment. Twenty-four hours later, the second hit was administered by intravenous injection of an anti-mouse MHC I monoclonal antibody (34-1-2S, IgG_2_a, Bio X Cell, USA) via the tail vein at a dose of approximately 1.0 mg/kg, completed within 60 s. Control mice received an equivalent volume of isotype control antibody (Rat IgG2a isotype control, Bio X Cell, USA). Where necessary, inhalation anesthesia with isoflurane was administered using an RWD R510 system (RWD Life Science, China), and fluid balance was maintained via subcutaneous injection of prewarmed PBS (10 mL/kg). Features of acute lung injury typically developed within 0.5-2 h post-second hit, and model validation was uniformly performed at 2 h post-injection.

To evaluate the therapeutic effect of the PEV@SP@EG nanosystem on TRALI, mice were randomly divided into four groups (*n* = 6 per group): Control group: Received intravenous injection of an equal volume of PBS. TRALI group: TRALI was induced using the two-hit model without any intervention. EGCG group: Free EGCG was administered concurrently with the first LPS injection, followed by a second dose 0.5 h before the second hit; the total dose of EGCG matched the equivalent content in the PEV@SP@EG group. PEV@SP@EG group: Free PEV@SP@EG nanosystem was injected at the time of the first LPS administration, and a second dose was given 0.5 h before the second hit (intravenous injection of PEV@SP@EG at 10 mg/kg). The dose-response data supporting this dose selection are summarized in Table S1. Mice in all groups were sacrificed 24 h after the second hit. Lung tissues were collected for blood gas analysis, wet/dry weight ratio (W/D) measurement, hematoxylin and eosin (H&E) staining, immunohistochemistry (IHC), enzyme-linked immunosorbent assay (ELISA), and flow cytometry to assess macrophage polarization and NETs formation in pulmonary tissue.

### 2.10 Measurement of arterial oxygen partial pressure (PaO_2_) and oxygenation index (PaO_2_/FiO_2_)

To assess oxygenation dysfunction in the murine TRALI model, arterial blood gas analysis was performed. Mice were anesthetized with sodium pentobarbital (100 mg/kg, intraperitoneally), and the common carotid artery was exposed for the collection of 0.3-0.5 mL of arterial blood using a heparinized syringe. PaO_2_ (mmHg) was immediately measured using an automated blood gas analyzer (ABL800 FLEX, Radiometer, Denmark). During the procedure, mice breathed spontaneously in ambient air (FiO_2_ = 0.21). The PaO_2_/FiO_2_ was calculated accordingly to reflect pulmonary oxygen exchange function.

### 2.11 Determination of lung W/D

To evaluate the degree of pulmonary edema, mice were euthanized at the end of the experiment, and the left lung was promptly excised. After gentle rinsing with sterile PBS and blotting to remove surface moisture, the lung was weighed to obtain the wet weight (W). The tissue was then dried in an oven at 60 °C for 48 h until a constant weight was achieved, and the dry weight (D) was recorded. The W/D was calculated as an indicator of tissue water content and the severity of pulmonary edema.

### 2.12 Determination of total protein (TP) content and total cell count in bronchoalveolar lavage fluid (BALF)

Following anesthesia with sodium pentobarbital (100 mg/kg, intraperitoneally), mice were euthanized. After exposing the trachea, a catheter was inserted, and the lungs were gently lavaged three times with 1 mL sterile PBS. The recovered fluids were pooled as the BALF sample. The BALF was first centrifuged at 300 ×g for 10 min at 4 °C to remove cells. The supernatant was collected for TP quantification, while the pellet was resuspended in PBS for total cell counting. TP content was measured using the BCA assay (BCA Protein Quantification Kit, P0012, Beyotime, China) according to the manufacturer's protocol. Absorbance was read at 562 nm using a microplate reader (SpectraMax iD3, Molecular Devices, USA), and protein concentration was calculated from a standard curve. Total cell count was determined manually using a hemocytometer after trypan blue staining, and the total and differential BALF cell-count data are summarized in Table S2.

### 2.13 H&E and Masson's trichrome staining of lung tissue

At the end of the experiment, the left lung tissue of each mouse was harvested and fixed in 4% paraformaldehyde (P0099, Beyotime, China) at 4 °C for 24 h. The samples were then dehydrated, cleared, embedded in paraffin, and sectioned at 4 μm using a microtome. The sections were subjected to standard H&E staining and Masson's trichrome staining.

H&E staining was used to evaluate alveolar structure, interstitial integrity, and inflammatory cell infiltration, while Masson's staining was employed to assess collagen deposition and the degree of fibrosis in the lung tissue. Lung injury scoring based on H&E sections was conducted in a blinded manner. The scoring criteria included alveolar wall thickening, hemorrhage, edema, neutrophil infiltration, and alveolar structural destruction, with each parameter graded on a scale from 0 to 4. The total score reflected the severity of lung injury. Masson's trichrome staining was performed using a commercial kit (G1340, Solarbio, China) according to the manufacturer's instructions.

Stained sections were imaged using an upright optical microscope (Axio Lab.A1, Zeiss, Germany), and alveolar integrity, interstitial thickening, inflammatory infiltration, and fibrosis were assessed at 200× and 400× magnifications.

### 2.14 *In vivo* biosafety assessment

To evaluate the *in vivo* safety of the PEV@SP@EG nanosystem, healthy mice were randomly divided into groups (*n* = 6 per group) and administered 200 μL of PBS, SP@EG nanoparticles, or PEV@SP@EG nanoparticles via tail vein injection (dose: 10 mg/kg) once daily. On day 7, mice were euthanized, and blood and major organs (liver, spleen, kidney, lung, and heart) were collected for subsequent biochemical analysis, hematological assessment, and histological examination.

### 2.15 Serum biochemical analysis

Cardiac blood was collected and centrifuged at 3000 rpm for 10 min at 4 °C to obtain serum. Alanine aminotransferase (ALT), aspartate aminotransferase (AST), blood urea nitrogen (BUN), and creatinine (CRE) levels were measured using corresponding assay kits: ALT Activity Assay Kit (MAK052, Sigma-Aldrich), AST Activity Assay Kit (MAK467, Sigma-Aldrich), BUN Assay Kit (MAS008, Sigma-Aldrich, USA), and Creatinine Assay Kit (MAK080, Sigma-Aldrich, UK).

### 2.16 Hematological analysis

Mouse blood samples were collected into EDTA-coated anticoagulant tubes and analyzed within 2 h using a veterinary hematology analyzer (Mindray BC-2800Vet, China) to measure red blood cell count (RBC), white blood cell count (WBC), hemoglobin concentration (HGB), and neutrophil count (NEU). All measurements were completed within 2 h to prevent cell lysis or aggregation.

### 2.17 Hemolysis assay

Peripheral blood (2 mL) was collected from healthy mice and centrifuged at 1000 ×g for 10 min at 4 °C to separate plasma. The red blood cells were washed three times with PBS buffer (pH 7.4, calcium- and magnesium-free; 10010023, Gibco, USA) and resuspended to prepare a 2% (v/v) erythrocyte suspension. Equal volumes of the erythrocyte suspension were incubated with varying concentrations of PEV@SP@EG solution (0.5, 5, 50, and 500 μg/mL) at 37 °C in a water bath for 2 h. The mixtures were then centrifuged at 1000 ×g for 5 min, and the absorbance of the supernatants at 540 nm (A_540_) was measured using a UV-visible spectrophotometer (UV-2600, Shimadzu, Japan). PBS-treated samples served as the negative control (0% hemolysis), and deionized water-treated samples as the positive control (100% hemolysis).

### 2.18 *In vivo* nanoparticle biodistribution tracking

Mice were intravenously injected with DiR-labeled SP@EG or PEV@SP@EG nanoparticles. *In vivo* fluorescence imaging was performed at multiple time points (3, 6, 12, 24, 48, and 72 h). At 24 h post-injection, major organs from the different injection groups and from TRALI model mice injected with PEV@SP@EG (heart, liver, spleen, lungs, kidneys) were harvested from each group for *ex vivo* fluorescence imaging. Fluorescence intensity was quantitatively analyzed using Living Image software, and the major-organ biodistribution data are summarized in Table S3.

### 2.19 IHC staining

Lung tissues were fixed in 10% neutral formalin (Servicebio, China) for 48 h, followed by routine paraffin embedding and sectioning at 4 μm thickness. After deparaffinization and rehydration, antigen retrieval was performed in citrate buffer (pH 6.0) at 95 °C for 30 min. Endogenous peroxidase activity was blocked with 3% H_2_O_2_ (v/v), followed by blocking of non-specific binding with 5% BSA (Beyotime, China) for 30 min. Sections were then incubated overnight at 4 °C with the following primary antibodies: anti-ZO-1 (ab96587, 1:200, Abcam, UK), anti-Occludin (ab167161, 1:200, Abcam, UK), anti-CD36 (ab124515, 1:250, Abcam, UK), anti-phospho-Fyn (ab184276, 1:200, Abcam, UK), and anti-myeloperoxidase (MPO) (ab208670, 1:1000, Abcam, UK). The following day, sections were incubated with an HRP-conjugated secondary antibody (ab205718, 1:500, Abcam) for 1 h, visualized with DAB substrate (ZSGB-BIO, China), and counterstained with hematoxylin. After dehydration and mounting, slides were scanned using whole-slide imaging. Quantitative analysis was performed using ImageJ software (v1.53, NIH, USA). Five random high-power fields (400×) per section were selected. The Color Deconvolution plugin was used to isolate the DAB channel and extract positive signals. Optical density (OD) was calculated based on grayscale values, and the mean optical density (MOD) of the positive area was used to represent protein expression levels.

### 2.20 Immunofluorescence staining

Immunofluorescence staining was performed to analyze immune cell markers in lung tissue and CD36/p-Fyn colocalization in M1-polarized macrophages. Paraffin-embedded sections were deparaffinized and rehydrated, followed by permeabilization with 0.3% Triton X-100 (Sigma-Aldrich, #T8787, USA) at room temperature for 10 min. Sections were then blocked with PBS containing 1% BSA (Solarbio, #A8020, China) for 60 min. After blocking, the following primary antibodies were applied and incubated overnight at 4 °C in the dark: anti-CD86 (ab119857, 1:200, Abcam, UK), rat anti-mouse CD36 (clone MF3; MCA2748GA, 1:200, Bio-Rad, UK), rabbit anti-phospho-Fyn (Tyr531, historically referenced as Tyr530; PA5-104756, 1:200, Invitrogen, USA), anti-MPO (ab208670, 1:200, Abcam, UK), and anti-Histone H3 (citrulline R17) (ab219407, 1:200, Abcam, UK). On the following day, sections were washed three times with PBS, each for 5 min. For CD36/p-Fyn dual immunofluorescence, CD36 was detected with goat anti-rat IgG H&L conjugated to Alexa Fluor 488 (ab150157, 1:1000, Abcam, UK), whereas p-Fyn was detected with goat anti-rabbit IgG H&L conjugated to Alexa Fluor 594 (ab150080, 1:1000, Abcam, UK). The samples were incubated with the secondary antibodies for 1 h at room temperature in the dark. Afterward, nuclei were counterstained with DAPI (#8961, 1:1000, Cell Signaling Technology, USA) at room temperature for 10 min. After three PBS washes, the samples were mounted and imaged using a confocal laser scanning microscope (CLSM, LSM880, Carl Zeiss, Germany).

### 2.21 Flow cytometry

To analyze the cellular subpopulations *in vitro* and in lung tissue, flow cytometry was performed. Mouse lung tissues were enzymatically digested using the Lung Dissociation Kit (#130-095-927, Miltenyi Biotec, Germany) and incubated at 37 °C for 30 min. The digested tissues were then passed through a 70 μm cell strainer to obtain single-cell suspensions. Red blood cells were lysed using RBC Lysis Buffer (#420301, BioLegend, USA), followed by two washes with PBS (300 ×g, 5 min each). The resulting cells were resuspended in FACS buffer (PBS with 2% fetal bovine serum [FBS]; #10099141, Gibco, USA) for staining. For each sample, 1 × 10^6^ cells were incubated with anti-CD16/CD32 antibody (Fc Block, #101320, 1:100, BioLegend) at room temperature for 10 min to block Fc receptors. Subsequently, fluorescently conjugated antibodies were added, and the cells were incubated at 4 °C in the dark for 30 min. The antibodies used included: anti-CD86 (ab119857, 1:200, Abcam, UK), anti-CD206 (ab270682, 1:200, Abcam, UK), anti-CD11c (ab254183, 1:200, Abcam, UK), anti-CD11b (ab128797, 1:200, Abcam, UK), anti-Siglec-F (155506, 1:200, BioLegend, USA), anti-Ly6G (ab238132, 1:200, Abcam, UK), and anti-F4/80 (ab16911, 1:200, Abcam, UK). Stained cells were analyzed using a BD FACSVerse flow cytometer (BD Biosciences, USA), with a minimum of 20,000 live-cell events collected per sample. Data were processed and analyzed using FlowJo software (v10.8.1, BD Biosciences).

### 2.22 Detection of pulmonary oxidative stress using a ROS fluorescent probe

The general ROS probe DCFH-DA (Beyotime, S0033S) was used to assess total ROS levels in lung tissue. Frozen lung sections were incubated with DCFH-DA working solution (10 μM) at 37 °C in the dark for 30 min. After incubation, sections were washed with PBS and counterstained with DAPI. Fluorescence intensity, indicating total ROS production, was observed using a Zeiss LSM 880 fluorescence microscope.

### 2.23 ELISA

To evaluate the expression levels of inflammatory cytokines and chemokines in lung tissue and cell culture supernatants, ELISAs were performed. Lung tissues were homogenized using a tissue homogenizer, and cell culture supernatants were centrifuged at 12,000 rpm for 10 min. The resulting supernatants were collected for analysis. All procedures were performed on ice, and repeated freeze-thaw cycles were avoided. Cytokine and chemokine levels were measured using the following commercial ELISA kits, following the manufacturers' instructions: IL-1β (ab197742, Abcam), TNF-α (ab208348, Abcam), IL-6 (ab100712, Abcam), IL-18 (ab216165, Abcam), CXCL1 (ab216951, Abcam), CXCL2 (ab272762, Abcam), CXCL8 (ab214030, Abcam), CXCL10 (ab260067, Abcam), IFN-γ (ab100689, Abcam), IL-10 (ab100697, Abcam), MPO (ab208670, Abcam), and dsDNA-HRP (ab117635, Abcam). Absorbance was measured at 450 nm using a microplate reader (SpectraMax iD5, Molecular Devices, USA). Each biological sample was assayed in technical triplicate. BALF supernatant total protein concentration was measured using the Bio-Rad DC method according to the manufacturer's instructions. BALF IgM levels were measured using a mouse IgM AlphaLISA kit (Cat# AL527S, PerkinElmer), and AlphaLISA signals were read on a Varioskan multimode plate reader equipped with AlphaScreen filters (Thermo Scientific). For BALF total and differential cell counts, the left main bronchus was cannulated after lung excision and bronchoalveolar lavage was performed. BALF cells were counted using a Neubauer chamber, cytospin preparations were stained with Diff-Quik, and BALF supernatants were stored at −80 °C before analysis.

### 2.24 RT-qPCR analysis

Total RNA was extracted from sorted cells using TRIzol™ reagent (Thermo Fisher Scientific, USA). RNA concentration was measured with a NanoDrop One spectrophotometer (Thermo Fisher Scientific, USA), and cDNA was synthesized using the HiScript® III RT SuperMix kit (Vazyme, China). Quantitative real-time PCR was performed with ChamQ SYBR qPCR Master Mix (Vazyme, China) on a QuantStudio™ 7 Flex system (Applied Biosystems, USA). Primer sequences are listed in Table S4. Relative gene expression levels were calculated using the 2^−ΔΔCt^ method, with GAPDH as the internal reference gene.

### 2.25 Western blot

Samples were lysed with RIPA buffer (R0010, Solarbio, China) supplemented with protease and phosphatase inhibitors (P1045, Beyotime, China), incubated on ice for 30 min, and centrifuged at 12,000 rpm for 10 min. The supernatants were collected, and protein concentrations were determined using the BCA assay (P0012, Beyotime, China). Equal amounts of TP (20-40 μg per sample) were loaded and separated on 10-12% SDS-PAGE gels. Proteins were then transferred to PVDF membranes (IPVH00010, Millipore, USA), followed by blocking in 5% non-fat milk in TBST at room temperature for 1 h. Membranes were incubated overnight at 4 °C with the following primary antibodies: CD86 (ab220188, 1:1000), iNOS (ab210823, 1:200), CD206 (ab300621, 1:1000), Fyn (ab184276, 1:1000), p-Fyn (Tyr530; ab182661, 1:1000), NF-κB p65 (ab16502, 1:1000), p-NF-κB p65 (ab76302, 1:1000), PAD4 (ab50332), citrullinated histone H3 (CitH3, ab219407, 1:1000), CD36 (ab124515, 1:1000), CD41 (ab181582), CD47 (ab214453), CD62P (ab59738), and GAPDH (ab8245, 1:5000) (all from Abcam, UK). The next day, membranes were washed three times with TBST (10 min each), then incubated at room temperature for 1 h with horseradish peroxidase (HRP)-conjugated secondary antibodies: goat anti-rabbit IgG-HRP (ab6721, 1:5000) or goat anti-mouse IgG-HRP (ab6789, 1:5000; Abcam, UK). Protein bands were visualized using ECL substrate (32106, Thermo Fisher Scientific, USA), and band intensities were quantified using ImageJ software (NIH, v1.53). Results were expressed as the ratio of the target protein to the internal reference (GAPDH) based on grayscale intensity. For co-immunoprecipitation, BMDM lysates were prepared with protease inhibitors, and cleared lysates were incubated overnight at 4 °C with an anti-CD36 antibody pre-bound to washed protein A/G magnetic beads. The resulting CD36 immunocomplexes were washed, eluted, and analyzed by Western blotting to detect interacting p-Fyn.

### 2.26 Isolation and culture of mouse bone marrow-derived macrophages (BMDMs) and neutrophils

Male C57BL/6 mice aged 6-8 weeks (purchased from Beijing Vital River Laboratory Animal Technology Co., Ltd.) were euthanized by cervical dislocation. Bilateral femurs and tibias were aseptically isolated, and bone marrow cells were flushed out using sterile PBS containing 2% FBS. The resulting cell suspension was filtered through a 70 μm cell strainer (Cat. No. 352350, Corning, USA) and centrifuged at 300 ×g for 5 min at 4 °C. The supernatant was discarded.

Macrophages: The collected bone marrow cells were resuspended in complete culture medium (high-glucose DMEM, C11995500BT, Gibco, USA) supplemented with 10% FBS and 1% penicillin-streptomycin. Recombinant mouse macrophage colony-stimulating factor (M-CSF, 20 ng/mL; Cat. No. 315-02, PeproTech, USA) was added, and cells were seeded into sterile 6-well plates at a density of approximately 5 × 105 cells per well. The cells were incubated at 37 °C with 5% CO_2_. On day 3, the medium was replaced with fresh complete medium containing M-CSF. By day 7, most cells had adhered and exhibited round or spindle-shaped morphology. These cells expressed CD11b^+^F4/80^+^ and were considered mature BMDMs, suitable for subsequent experiments [18].

Neutrophils: Bone marrow cells were processed using a Mouse Neutrophil Isolation Kit (Cat. No. 130-097-658, Miltenyi Biotec, Germany) according to the manufacturer's instructions. Neutrophils (PMNs) were isolated by magnetic negative selection. The purified neutrophils were resuspended in phenol red-free RPMI 1640 medium (SH30809.01, HyClone, USA) supplemented with 2% FBS and 1% penicillin-streptomycin. Cell concentration was adjusted to 1 × 10^6^ cells/mL. Cells were then seeded onto 12-well plates with glass coverslips and cultured short-term at 37 °C in a 5% CO_2_ incubator [18].

### 2.27 Lentiviral transduction

Mouse-derived *Cd3*6-shRNA and Fyn-shRNA were purchased from Sigma-Aldrich. HEK293T cells (Cat. No. BFN60810479, ATCC) were cultured in DMEM supplemented with 10% FBS (Gibco) at 37 °C in a humidified atmosphere containing 5% CO_2_. Lentiviral interference plasmids encoding *Cd3*6-shRNA or Fyn-shRNA (sh-*Cd36*-luc, sh-Fyn-luc), as well as a negative control plasmid (sh-NC-luc), were co-transfected with packaging helper plasmids into HEK293T cells. Plasmid construction and viral packaging were performed by Sangon Biotech (Shanghai, China). Viral supernatants were collected, validated, expanded, and purified to obtain high-titer lentiviral stocks (∼5 × 10^6^ TU/mL). The target sequences are listed in Table S5. Macrophages in the logarithmic growth phase were seeded into 6-well plates at a density of 3 × 10^5^ cells/well. When cell confluency reached 70-90%, lentiviral transduction was carried out using infection medium supplemented with polyethylene glycol enhancer. Specifically, lentivirus was added at a multiplicity of infection (MOI) of 10, along with polybrene (5 μg/mL; TR-1003, Merck, USA). After 4 h of infection, an equal volume of culture medium was added to dilute the viral suspension, and cells were incubated for an additional 24 h before the medium was replaced with fresh complete medium. At 48 h post-infection, transduction efficiency was assessed using a luciferase reporter assay. Cells were then subjected to puromycin selection (2-5 μg/mL; A1113803, Gibco, USA) until all uninfected cells were eliminated, thereby establishing stable knockdown cell lines. The knockdown efficiency was further validated by RT-qPCR analysis of *Cd36* and Fyn mRNA expression. The sh-CD36-2 and sh-Fyn-1 sequences were selected for subsequent cell transfection experiments.

### 2.28 Treatment and grouping of mouse BMDMs

Following the preparation and maturation of BMDMs as described above, cells were seeded into 6-well plates at a density of 5 × 10^5^ cells/well. After overnight adherence, cells were treated and grouped as follows to simulate the inflammatory microenvironment observed in TRALI and to investigate the role of the CD36/Fyn signaling axis in M1 polarization: Control group (Ctrl): Cells were incubated with an equal volume of serum-free complete DMEM for 24 h. M1 polarization group (LPS + IFN-γ): Cells were treated with LPS (100 ng/mL) and IFN-γ (20 ng/mL) for 24 h to induce M1 polarization. CD36 overexpression (oe-CD36) group (M1 + oe-CD36): BMDMs were first transfected with an oe-CD36 plasmid. After confirming stable overexpression, cells were treated with LPS + IFN-γ for 24 h. CD36 knockdown (sh-CD36) group (M1 + sh-CD36): BMDMs were transduced with lentivirus encoding CD36 shRNA, followed by LPS + IFN-γ stimulation for 24 h. CD36/Fyn axis inhibition group (M1 + oe-CD36 + sh-Fyn or PP2): Based on oe-CD36, BMDMs were either co-transfected with a Fyn shRNA plasmid or pretreated with the Fyn inhibitor PP2 (10 μM) 1 h before LPS + IFN-γ stimulation for 24 h. NF-κB inhibition group (M1 + oe-CD36 + Bay11-7082): Following oe-CD36 and LPS + IFN-γ treatment, cells were pretreated with the NF-κB inhibitor Bay11-7082 (5 μM) 1 h before stimulation to assess downstream inflammatory signaling. After treatment, cells were collected for Western blot, RT-qPCR, and ROS probe staining to evaluate CD36/Fyn signaling and NF-κB pathway activation. The culture supernatants were harvested as conditioned medium (CM) for subsequent neutrophil functional assays and ELISA analysis of chemokines, including CXCL1, CXCL2, CXCL8, and CXCL10.

### 2.29 Treatment and grouping of neutrophils

Mouse bone marrow-derived neutrophils (neutrophils, PMNs) of high purity were isolated as previously described and seeded at a density of 1 × 10^6^ cells/mL into glass-bottom culture dishes or Transwell inserts. Cells were incubated in phenol red-free RPMI-1640 medium. To evaluate the effects of BMDM-derived CM and the CD36/Fyn-CXCL axis on neutrophil migration and NETosis, the following treatment groups were established: Baseline control group (PMNs + Ctrl-CM): Neutrophils were incubated with CM derived from untreated BMDMs, diluted with RPMI-1640 to an equal ratio. M1-CM group (PMNs + M1-CM): Neutrophils were exposed to CM from LPS + IFN-γ-induced M1-polarized BMDMs to assess the impact of inflammatory CM on neutrophil activation and NET formation. oe-CD36 CM group (PMNs + oe-CD36-M1-CM): Neutrophils were treated with CM collected from oe-CD36 BMDMs (treated with LPS + IFN-γ) to evaluate the pro-migratory and pro-NETotic effects mediated by elevated CXCL secretion. CD36/Fyn inhibition CM group (PMNs + oe-CD36 + sh-Fyn-M1-CM or + PP2-M1-CM): Neutrophils were incubated with CM from BMDMs in which Fyn was either genetically silenced or pharmacologically inhibited, to confirm the role of Fyn in CD36-mediated pro-inflammatory and chemotactic responses. NF-κB inhibition CM group (PMNs + oe-CD36 + Bay11-7082-M1-CM): Neutrophils were incubated with CM from BMDMs treated with both oe-CD36 and LPS + IFN-γ, along with the NF-κB inhibitor Bay11-7082, to explore the involvement of downstream NF-κB signaling in CXCL release and NETosis. CXCR2 antagonist group (PMNs + M1-CM + SB225002): Neutrophils were pretreated for 30 min with the CXCR2 antagonist SB225002 (100 nM) before incubation with M1-CM or oe-CD36-M1-CM, aiming to block the CXCL-CXCR2 axis and assess whether neutrophil migration and NET formation could be reversed. After 2 h of incubation, neutrophil migration and NETosis were assessed using Transwell migration assays, Sytox Green staining, and MPO-DNA ELISA, respectively.

### 2.30 EGCG drug intervention and grouping

To investigate the role of EGCG in regulating macrophage polarization and NETosis formation, an *in vitro* M1 polarization model of macrophages was established with the following experimental groups: Control group: Untreated macrophages serving as the baseline control. M1 group: Macrophages were stimulated with LPS (100 ng/mL) and IFN-γ (20 ng/mL) for 24 h to induce M1 polarization. M1 + EGCG group: EGCG (50 μM) was added concurrently with LPS and IFN-γ stimulation for 24 h to assess its regulatory effects on macrophage polarization. M1 + EGCG + oe-CD36 group: oe-CD36 plasmids were transfected into macrophages before LPS/IFN-γ stimulation in the presence of EGCG to further evaluate the role of CD36 in EGCG-mediated modulation. For the neutrophil NETosis assay, CM were collected from the macrophages in each group and used to stimulate neutrophils for 6 h, followed by evaluation of NET formation.

### 2.31 Transwell migration assay

A 24-well Transwell system (8 μm pore size, Corning) was employed. The lower chambers were filled with 600 μL of CM derived from different treatment groups. Neutrophils (1 × 10^6^ cells in 100 μL RPMI-1640 without serum) were seeded in the upper chambers and incubated for 2 h. Non-migrated cells on the upper membrane surface were removed, and migrated cells were fixed with methanol and stained with crystal violet. Migrated cells were imaged using an inverted microscope and quantified.

### 2.32 Intracellular ROS fluorescence staining

BMDMs were washed with PBS and incubated with the ROS-sensitive probe DCFH-DA (20 μM, S0033S, Beyotime, China) at 37 °C for 30 min in the dark. After staining, cells were washed three times with PBS. Fluorescence was observed and recorded using a CLSM (LSM 880, Zeiss, Germany) with excitation at 488 nm and emission at 525 nm. Quantitative analysis of fluorescence intensity was performed using ImageJ software, and representative images were presented.

### 2.33 Sytox Green staining to assess neutrophil extracellular DNA release associated with NET formation

Neutrophils were seeded into glass-bottom dishes at a density of 1 × 10^6^ cells per dish and co-incubated with different CM for 4 h. Sytox Green dye (5 μM, S7020, Thermo Fisher) was then added for 10 min, followed by nuclear staining with DAPI (1 μg/mL, Beyotime). Excess dye was removed by a single PBS wash. Green fluorescence signals, indicating extracellular DNA release associated with NETs formation, were observed using a CLSM (LSM 880, Zeiss, Germany) with an excitation wavelength of 488 nm. Fluorescence intensity was quantified per field of view using ImageJ software.

### 2.34 Cell cytotoxicity and viability assay

BMDMs were seeded in 6-well plates at a density of 5 × 105 cells per well and incubated overnight in DMEM medium (C11995500BT, Gibco, USA) supplemented with 10% FBS and 1% penicillin-streptomycin to allow cell attachment. The following day, the medium was replaced, and cells were treated with varying concentrations of PEV@SP@EG nanoparticles (50, 100, and 200 μg/mL), followed by incubation at 37 °C in a 5% CO_2_ atmosphere for 24 h.

Cell viability was assessed using a Calcein-AM/PI dual-staining live/dead assay kit (C2015S, Beyotime, China). The staining solution was prepared using serum- and antibiotic-free medium containing 2 μM Calcein-AM and 4 μM PI. After 20 min of incubation in the dark, cells were gently washed once with PBS. Fluorescence imaging was performed using a CLSM (LSM 880, Zeiss, Germany). Green fluorescence (live cells) was detected at 488 nm excitation, and red fluorescence (dead cells) at 561 nm. Cell-viability measurements were obtained from three independent biological experiments and quantified using image-analysis software.

### 2.35 Apoptosis detection

Apoptosis was evaluated using the Annexin V-FITC/PI dual-staining assay kit (556547, BD Biosciences, USA). Following treatment, BMDMs were washed twice with PBS, harvested, and resuspended in binding buffer at a concentration of 1 × 10^6^ cells/mL. To 100 μL of cell suspension, 5 μL each of Annexin V-FITC and PI was added, and samples were incubated in the dark for 15 min. After staining, 400 μL of binding buffer was added, and samples were immediately analyzed using a flow cytometer (FACSCanto II, BD Biosciences, USA). The percentage of apoptotic cells was quantified using FlowJo software.

### 2.36 Confocal microscopy for cellular uptake analysis

To investigate the intracellular uptake behavior of nanoparticles, PEVs were labeled with the green fluorescent lipophilic dye DiO (V22886, Invitrogen, USA), and EGCG was labeled with the red fluorescent dye TAMRA (5(6)-TAMRA SE, 89606-007, Sigma-Aldrich). After preparation, the fluorescently labeled PEV@SP@EG nanoparticles were co-incubated with BMDMs for 24 h. Following incubation, the cells were washed three times with PBS, stained with DAPI (1 μg/mL, C1002, Beyotime, China) for 5 min to label the nuclei, and washed again with PBS before being mounted onto slides. Fluorescence distribution within the cells was observed using a CLSM. Excitation wavelengths were set at 488 nm for DiO (green), 561 nm for TAMRA (red), and 405 nm for DAPI (blue), and colocalization of the nanoparticles within the cells was recorded.

### 2.37 Preparation of single-cell suspensions from lung tissue

Lung tissue samples from both the TRALI group and the control group (one sample per group) were collected under sterile conditions. Tissues were enzymatically digested at 37 °C for 30 min using a solution containing 0.2 mg/mL collagenase IV and 0.1 mg/mL DNase I (Sigma-Aldrich, USA). The resulting digests were filtered through a 70 μm cell strainer, and red blood cells were removed using ACK lysing buffer (Thermo Fisher, USA). The digestion was terminated by adding 10% FBS (Gibco, USA). The resulting single-cell suspension was counted using a hemocytometer, and cell viability was assessed to ensure a viability rate above 90%.

### 2.38 Single-cell RNA sequencing (scRNA-seq) and data analysis

Single-cell library construction was performed using the Chromium Single Cell 3′ Reagent Kit v3.1 (10x Genomics, USA), with a target capture of 7500 cells per sample, following the manufacturer's protocol. The resulting libraries were sequenced on the HiSeq X Ten platform (Illumina, USA) using 150 bp paired-end reads, with a target sequencing depth of approximately 50,000 reads per cell. Raw FASTQ files were processed using Cell Ranger software (v6.1.2, 10x Genomics) for deduplication and alignment to the mouse reference genome GRCm38, generating expression matrices for downstream analysis. Data quality control and processing were conducted using the Seurat package (v4.3.0). High-quality cells were retained by filtering for those with more than 500 detected genes and mitochondrial gene content below 10%. The merged expression matrix was log-normalized (scale factor set to 10,000), and the top 2000 highly variable genes were selected for principal component analysis (PCA). The first 30 principal components (PCs) were used for subsequent analyses. To mitigate batch effects across samples, the Harmony package (v0.1.1) was applied for batch correction. Clustering analysis was conducted using the Louvain algorithm implemented via the FindNeighbors and FindClusters functions (resolutio*n* = 0.7). Two-dimensional visualization was performed using the RunUMAP function. Cell subtype annotation was based on the CellMarker database (http://bio-bigdata.hrbmu.edu.cn/CellMarker/) and previously reported marker genes. Major identified cell types included endothelial cells, fibroblasts, B cells, neutrophils, macrophages, T cells, monocytes, and natural killer (NK) cells. Cell composition proportions were summarized and visualized descriptively using the table and prop.table functions in R, grouped by orig.ident. Because the scRNA-seq dataset included only one biological sample per group, these proportions were treated as exploratory descriptive results rather than as sample-level statistical inference.

### 2.39 Module scoring analysis

Immune polarization states were evaluated by calculating gene module scores using the AddModuleScore function. The M1 module included the following genes: Nos2, Il1b, Tnf, Cxcl10, Cd86, Stat1, Socs3, Nfkb1, Ccl5, and Il6. The M2 module comprised Arg1, Mrc1, Chi3l3, Cd163, Retnla, Il10, Tgfbi, Trem2, Fizz1, and Clec10a. Violin plots were generated using VlnPlot to descriptively visualize module score distributions across samples. Because only one biological sample was included per group, sample-level significance testing was not performed for module scores, and these results were used only for exploratory analysis and subsequent mechanistic validation.

### 2.40 Macrophage subset analysis and functional annotation

Macrophages were extracted using the subset function (Idents = “Macrophages”), followed by a second round of PCA and UMAP dimensionality reduction. Clustering was performed with a resolution parameter set to 0.6, identifying 15 distinct subpopulations. Based on marker gene expression profiles, these subclusters were functionally annotated as M1_inflammatory, M2_regulatory, M2_tissue_repair, and IFN_response. Proportional distributions of these functional subsets were descriptively visualized using bar plots, without sample-level statistical comparison.

### 2.41 Differential expression and functional enrichment analysis

To explore potential transcriptional-change clues in macrophages under TRALI conditions, expression changes between macrophages from the TRALI and sham samples were analyzed using the FindMarkers function in Seurat (min.pct = 0.25, logfc.threshol*d* = 0.25). Because the scRNA-seq dataset included only one biological sample per group, the FindMarkers output was used only to prioritize candidate expression-change genes and indicate expression-change direction, rather than to define statistically significant group-level DEGs. Volcano plots were generated using the EnhancedVolcano package (v1.18.0). GO and KEGG enrichment analyses were performed using the clusterProfiler package (v4.6.2), with annotation from org.Mm.eg.db, to provide exploratory functional clues for subsequent validation.

### 2.42 Gene Co-expression correlation analysis

Expression matrices for *Cd36* and selected M1 marker genes (Cd86, Cd11c, Il1b, Ccl3, Ccl5, Stat1, Tnf, Nfkb1) in macrophages were extracted using the FetchData function. Correlation analysis and scatter plots were generated with the ggscatter function from the ggpubr package, using Pearson's correlation coefficient (cor.metho*d* = “pearson”). Correlation coefficients were displayed as exploratory co-expression information and were not interpreted as sample-level statistical validation or causal evidence.

### 2.43 Statistical analysis

All experimental data were presented as mean ± standard deviation (Mean ± SD). Statistical analyses were performed using GraphPad Prism version 10.0 (GraphPad Software, USA). Comparisons between two groups were conducted using two-tailed unpaired *t*-tests. One-way or two-way ANOVA was used according to the number of experimental factors. Dunnett's multiple-comparisons test was used when treatment groups were compared with a prespecified control, whereas Tukey's post-hoc test was used for all pairwise comparisons. For non-normally distributed two-group data, the Mann-Whitney *U* test was used. A *p*-value < 0.05 was considered statistically significant. Significance levels were denoted as follows: *p < 0.05, **p < 0.01, ***p < 0.001, ****p < 0.0001. For quantitative cell-based experiments, data were obtained from three independent biological experiments, and each plotted value represented one independent experiment. For the time-course ELISA, each chemokine was analyzed separately across five independent time-point groups (0, 6, 12, 24, and 36 h) using an ordinary one-way ANOVA followed by Tukey's multiple-comparisons test; no repeated-measures structure was assumed. Biologically independent animals were considered the experimental units for all *in vivo* statistical analyses, and technical or assay-level repeated measurements were not treated as independent biological replicates. For the four-group *in vivo* experiment, six biologically independent mice were analyzed per group (total *N* = 24). Sensitivity was evaluated for a fixed-effects omnibus one-way ANOVA using the noncentral *F* distribution at *α* = 0.05. Endpoint-specific observed *η*^*2*^, Cohen's *f*, and retrospective power were calculated from the animal-level quantitative data. For cell-based designs with three independent biological experiments per group, design-level sensitivity was evaluated using exact noncentral *t* or *F* distributions at *α* = 0.05 according to the number of groups. Measurement-specific observed effect sizes and retrospective power estimates were interpreted descriptively.

## 3 Results

### 3.1 Successful Establishment of the TRALI animal model exhibiting typical features of acute lung injury

To establish a stable model of TRALI, a “two-hit” protocol was employed. H&E staining was first used to assess pulmonary histopathology. Lung tissues from the TRALI group exhibited pronounced alveolar wall thickening, accompanied by marked hemorrhage, interstitial edema, and extensive neutrophil infiltration. In contrast, the control group displayed intact alveolar architecture, sparse interstitium, and minimal inflammatory cell infiltration (Fig. S1A–B). To further evaluate pulmonary vascular permeability, protein levels in BALF were quantified. The TRALI group showed a significant increase in BALF protein concentration (Fig. S1C), indicating disruption of the alveolar-capillary barrier. Consistently, the lung W/D—a marker of pulmonary edema—was also markedly elevated in the TRALI group, reflecting exacerbated fluid retention in lung tissues (Fig. S1D).

IHC staining revealed disrupted expression and mislocalization of the alveolar epithelial adherens junction protein E-cadherin and the tight junction protein ZO-1 in TRALI lungs, further indicating structural damage to the alveolar-capillary barrier (Fig. S1E–F). To assess local pulmonary inflammation, cytokine levels in BALF were measured. ELISA results demonstrated significantly increased concentrations of pro-inflammatory cytokines TNF-α and IL-1β, alongside a notable decrease in the anti-inflammatory cytokine IL-10 in the TRALI group (Fig. S1G), suggesting a robust pro-inflammatory cascade and immune imbalance triggered by TRALI. Moreover, Masson's trichrome staining revealed enhanced collagen deposition in the alveolar interstitium of the TRALI group (Fig. S1H), indicating early onset of pulmonary tissue remodeling. Blood gas analysis further showed that arterial PaO_2_ and PaO_2_/FiO_2_ were decreased in the TRALI group (Fig. S1I–J), further supporting successful model induction.

Collectively, these findings confirm the successful induction of a TRALI model that recapitulates key pathological features of acute lung injury, rendering it suitable for subsequent mechanistic and interventional investigations.

### 3.2 Exploratory single-cell transcriptomic atlas suggests changes in pulmonary immune-cell composition and macrophage inflammatory phenotype in TRALI

**Fig. 1:**
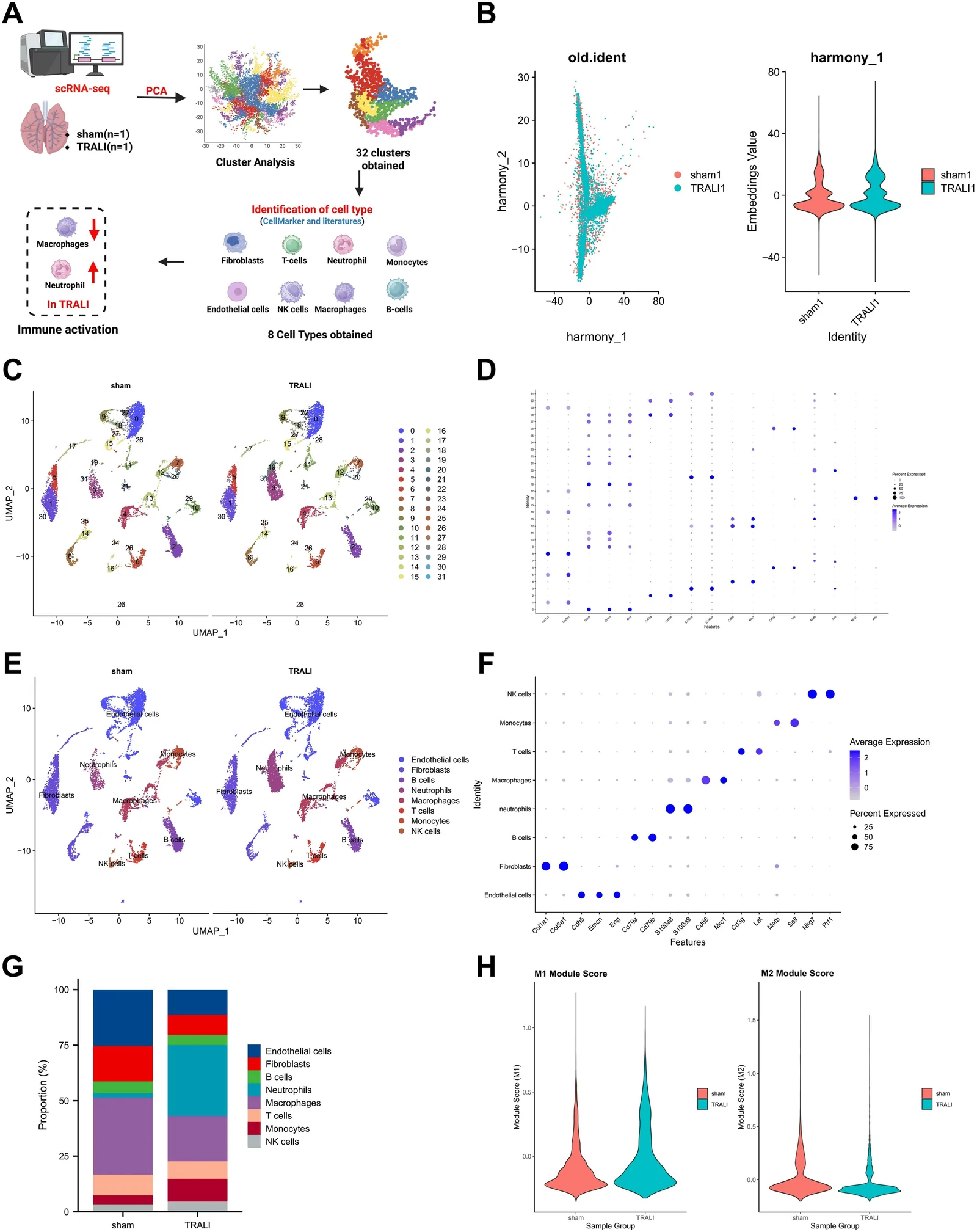
Exploratory scRNA-seq Analysis Maps the Cellular Composition Atlas of TRALI Lung Tissue. Note: (A) Schematic diagram of the scRNA-seq analysis workflow; (B) Dimensionality reduction plots before and after Harmony integration and embedding violin plots showing correction of technical differences between samples; (C) UMAP clustering plot shown by sham and TRALI samples, where each dot represents a single cell, and colors indicate cluster identity; (D) Dot plot of marker gene expression across clusters; dot size reflects the proportion of cells expressing the gene, and color intensity indicates the average expression level; (E) UMAP plot annotated by major cell types, showing the distribution of cell types in sham and TRALI groups; (F) Dot plot of marker gene expression for major cell types supporting cell type annotation; (G) Bar plot of the proportion of major cell types across different groups; (H) Violin plots of M1 and M2 gene module scores based on immune-related markers, comparing the distribution of polarization scores between sample groups.

In this study, scRNA-seq based on the 10x Genomics platform was employed to analyze lung tissues from TRALI model mice (*n* = 1) and sham controls (*n* = 1), as an exploratory sequencing analysis aiming to characterize cellular composition changes under TRALI (Fig. 1A). Because these scRNA-seq data were derived from one biological sample per group, this analysis was used primarily to construct a descriptive cellular atlas and generate mechanistic clues rather than to support independent sample-level statistical inference. Following stringent quality control, 46,823 high-quality cells were retained for downstream analysis. The number of transcripts per cell (nCount_RNA), the number of detected genes per cell (nFeature_RNA), and the percentage of mitochondrial gene expression (percent.mt) all fell within acceptable ranges (Fig. S2A). PCA revealed distributional differences between samples. After integration using the Harmony algorithm, technical differences between samples were partially corrected, supporting subsequent descriptive clustering and cell-type annotation (Fig. 1B; Fig. S2B). The heatmap of top-ranking genes from the first six PCs (Fig. S2C) was further utilized to guide downstream clustering and dimensionality reduction analyses.

Based on the above, the top 2000 highly variable genes were selected to construct the expression matrix, and clustering was performed using the Louvain algorithm (resolutio*n* = 0.7), yielding 32 distinct cell clusters. UMAP projection showed broadly comparable cluster distributions between the sham and TRALI samples after integration, supporting descriptive downstream annotation (Fig. 1C). By referencing the CellMarker database and examining the distribution of canonical marker genes (Fig. 1D–F), the clusters were annotated into eight major cell populations, including endothelial cells, fibroblasts, B cells, neutrophils, macrophages, T cells, monocytes, and NK cells (Fig. 1E).

Further analysis of cell proportions descriptively suggested an increased frequency of neutrophils and a decreased proportion of macrophages in the TRALI sample, suggesting potential immune-cell compositional remodeling (Fig. 1G). In addition, M1 and M2 immune gene modules were constructed using the AddModuleScore function to assess polarization states across samples. The module-score distributions suggested higher M1-related transcriptional activity and lower M2-related transcriptional activity in macrophages from the TRALI sample, indicating a potential shift toward a proinflammatory M1-like state (Fig. 1H). It should be emphasized that these module-score results were used only as candidate clues for subsequent mechanistic studies, and their biological significance requires further validation using independent animal samples and *in vitro* functional experiments.

Taken together, this exploratory single-cell transcriptomic atlas preliminarily suggested changes in pulmonary immune-cell composition, relative enrichment of neutrophils, and enhanced pro-inflammatory transcriptional programs in macrophages in TRALI lungs, providing candidate evidence for subsequent mechanistic validation of the CD36/Fyn-CXCL-NETosis axis.

### 3.3 Exploratory Macrophage Subcluster Analysis Suggests Enhanced Pro-Inflammatory Transcriptional States in TRALI lung tissue

**Fig. 2:**
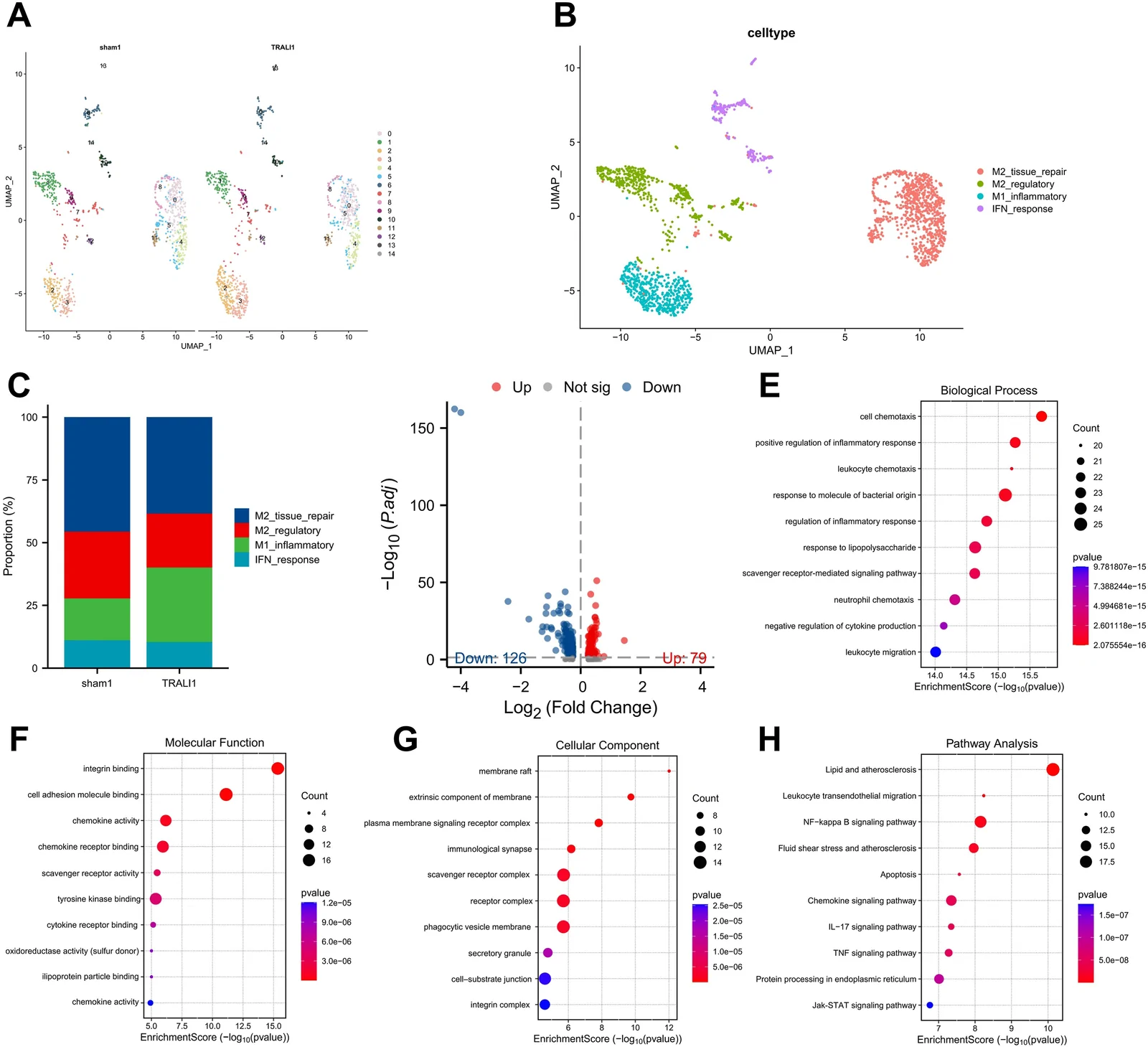
Exploratory Macrophage Subcluster Analysis Suggests Enhanced Pro-Inflammatory Transcriptional States in TRALI Lung Tissue. Note: (A) UMAP plot showing the distribution of 15 macrophage subclusters in the sham and TRALI groups; (B) Subclusters were annotated based on marker genes into four functional categories: M1_inflammatory, M2_regulatory, M2_tissue_repair, and IFN_response; (C) Bar plot illustrating the proportion of each macrophage subtype in the sham and TRALI groups; (D) Volcano plot of candidate expression-change genes in macrophages between the TRALI and sham groups; (E) GO enrichment analysis of BP; (F) GO enrichment analysis of MF; (G) GO enrichment analysis of CC; (H) KEGG pathway enrichment analysis.

Based on the previous UMAP clustering, we extracted macrophages for secondary clustering analysis and identified 15 transcriptionally distinct subpopulations for exploratory functional annotation (Fig. 2A). According to the expression patterns of differentially expressed marker genes, these subpopulations were classified into four functional states: M1_inflammatory, M2_regulatory, M2_tissue_repair, and IFN_response (Fig. 2B). Descriptive visualization of subset proportions suggested a relative increase in M1_inflammatory macrophages in the TRALI sample, accompanied by a lower proportion of M2-related subsets (Fig. 2C), indicating a potential proinflammatory polarization trend under TRALI conditions.

To further characterize the transcriptional differences between macrophages from the TRALI and sham groups, exploratory expression-change analysis identified 205 candidate genes with altered expression direction, including 79 upregulated and 126 downregulated genes (Fig. 2D). GO enrichment analysis suggested that these candidate genes were mainly involved in BP, such as “cell chemotaxis,” “regulation of inflammatory response,” “response to lipopolysaccharide,” and “cell migration” (Fig. 2E). The MF were enriched in categories including “integrin binding,” “chemokine activity,” and “receptor-ligand activity” (Fig. 2F), while the associated CC were primarily localized to the “plasma membrane protein complex,” “extracellular matrix,” and “endoplasmic reticulum-related structures” (Fig. 2G). KEGG pathway analysis further suggested enrichment trends in immune-inflammatory signaling cascades, including the “chemokine signaling pathway,” “IL-17 signaling pathway,” “TNF signaling pathway,” and “NF-κB signaling pathway” (Fig. 2H).

Taken together, exploratory macrophage subcluster analysis suggested a potential enhancement of pro-inflammatory macrophage transcriptional states in TRALI lung tissue, accompanied by trends toward chemokine- and inflammation-related pathway activation. These findings provided candidate clues for subsequent functional validation of the CD36/Fyn-CXCL-NETosis axis.

### 3.4 CD36 deficiency or knockdown suppresses M1 polarization and restores immune homeostasis

Previous studies have demonstrated that CD36 plays a pivotal role in regulating macrophage pro-inflammatory polarization and amplifying inflammatory responses [19,20]. Therefore, we next examined its expression pattern and potential function within the TRALI context. By projecting *Cd36* expression onto the UMAP embedding of macrophage subsets, we observed that the *Cd36* signal was mainly distributed in the M1_inflammatory cluster, whereas partial expression was detected in the IFN_response subset. In contrast, the M2_regulatory and M2_tissue_repair subsets exhibited relatively weaker *Cd36* signals. These descriptive findings suggested that *Cd36* may be associated with M1-like pro-inflammatory macrophage features within the TRALI context (Fig. S3A). We subsequently descriptively assessed the single-cell-level co-expression pattern between CD36 and canonical macrophage marker genes. The results showed that *Cd36* tended to be co-expressed with Cd86, Ccl11, Il1b, Ccl3, Ccl5, Stat1, Tnf, and Nfkb1 (Fig. S3B), supporting an exploratory association with inflammatory transcriptional features rather than sample-level statistical inference. To clarify the regulatory role of CD36 in macrophage polarization, we generated oe-CD36 and sh-CD36 models and included an empty vector control (oe-NC) for comparison (Fig. S3C). Western blot analysis revealed markedly increased expression of CD86 and iNOS in the oe-CD36 group, whereas the sh-CD36 group exhibited reduced CD86 and iNOS levels, indicating that CD36 promotes M1 polarization (Fig. S3D). Flow cytometric analysis further confirmed these findings: compared with the oe-NC group, the proportion of CD86^+^ macrophages increased in the oe-CD36 group, while the sh-CD36 group showed a significant reduction in CD86^+^ cells (Fig. S3E).

ELISA results showed that proinflammatory cytokines—including IL-1β, TNF-α, IL-6, and IL-18—were elevated in the oe-CD36 group, whereas their levels decreased in the sh-CD36 group, demonstrating that CD36 upregulation enhances inflammatory activation, whereas its silencing attenuates the proinflammatory phenotype (Fig. S3F). ROS fluorescence staining further revealed markedly increased ROS production in the oe-CD36 group, indicating an intensified oxidative burst; conversely, ROS fluorescence was significantly reduced in the sh-CD36 group, suggesting that CD36 expression influences inflammatory metabolic activity (Fig. S3G).

Collectively, these findings demonstrate that CD36 plays a crucial role in regulating macrophage M1 polarization and inflammatory cytokine production, and that modulation of CD36 expression effectively alters immune-polarization states.

### 3.5 CD36 Engages Fyn Signaling during proinflammatory polarization

**Fig. 3:**
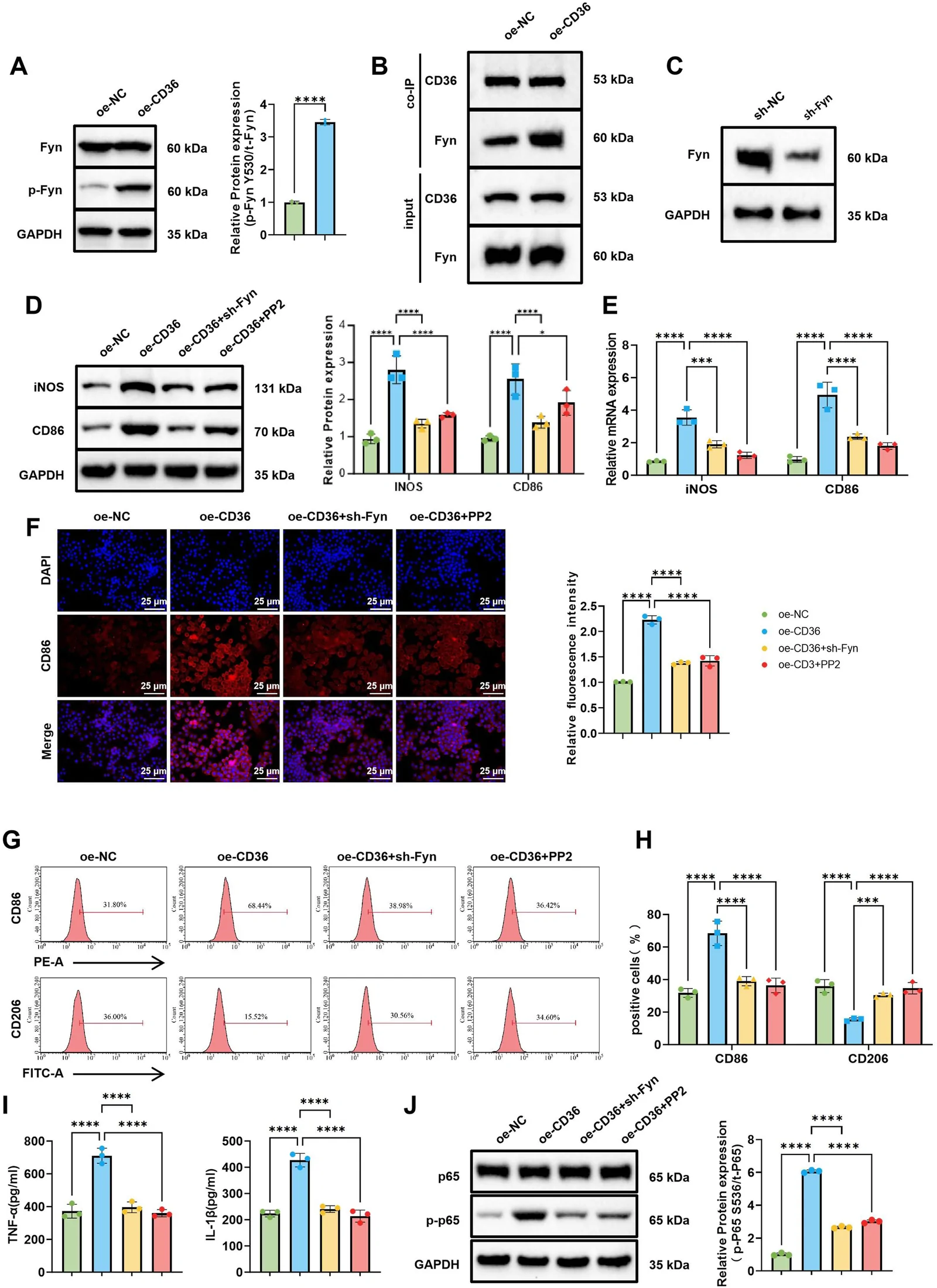
CD36 Promotes Macrophage M1 Polarization and Proinflammatory Cytokine Secretion via Fyn Signaling. Note: (A) Western blotting and densitometric analysis of p-Fyn (Tyr530)/Fyn in oe-NC and oe-CD36 BMDMs; (B) co-immunoprecipitation analysis of the interaction between CD36 and Fyn in oe-NC and oe-CD36 BMDMs, with input controls; (C) Western blot verification of Fyn knockdown in sh-NC and sh-Fyn BMDMs; (D) Western blotting and densitometric analysis of iNOS and CD86 in oe-NC, oe-CD36, oe-CD36 + sh-Fyn, and oe-CD36 + PP2 groups; (E) RT-qPCR analysis of iNOS and CD86 mRNA expression in the same groups; (F) immunofluorescence staining and quantitative analysis of CD86 (red), with DAPI nuclear counterstaining (blue); scale bar = 25 μm; (G-H) flow cytometric analysis and quantification of CD86^+^ and CD206+ macrophages; (I) ELISA quantification of TNF-α and IL-1β in culture supernatants; (J) Western blotting and densitometric analysis of p-NF-κB p65/total NF-κB p65. Quantitative cell-based data were obtained from three independent biological experiments (*n* = 3 per group), with each plotted value representing one independent experiment. *p < 0.05; **p < 0.01; ***p < 0.001; ****p < 0.0001.

To investigate the downstream signaling mechanisms of CD36 during macrophage M1 polarization, we examined the phosphorylation status of Fyn, a member of the Src family of tyrosine kinases. Western blot analysis showed that oe-CD36 increased phosphorylation at Fyn Tyr530 without altering total Fyn expression, demonstrating a CD36-associated change in Fyn phosphorylation (Fig. 3A). Co-immunoprecipitation was then performed in BMDMs to verify the physical interaction between CD36 and Fyn. The results showed that CD36 interacted with Fyn and that CD36 overexpression significantly enhanced the CD36-Fyn interaction (Fig. 3B).

To further validate this mechanism, oe-CD36 BMDMs were subjected to either sh-Fyn, whose knockdown efficiency was confirmed by Western blot (Fig. 3C), or treatment with the Src family kinase inhibitor PP2. Western blot results showed that both interventions markedly reduced protein levels of the M1 markers CD86 and iNOS in the oe-CD36 context (Fig. 3D). RT-qPCR confirmed a corresponding decline in CD86 and iNOS mRNA expression (Fig. 3E).

Immunofluorescence analysis further demonstrated that oe-CD36 increased the number of CD86^+^ cells, whereas sh-Fyn and PP2 treatment reversed this effect (Fig. 3F). Flow cytometry showed that the oe-CD36 group exhibited an elevated proportion of CD86^+^ macrophages and a reduced proportion of CD206^+^ cells. In contrast, co-treatment with sh-Fyn or PP2 significantly decreased the M1 population and restored the M2 phenotype (Fig. 3G and H). ELISA results indicated that TNF-α and IL-1β secretion were markedly upregulated in the oe-CD36 group, while both sh-Fyn and PP2 significantly suppressed their release (Fig. 3I). In addition, oe-CD36 activated the NF-κB signaling pathway, as evidenced by increased p-p65 levels, which were notably downregulated upon Fyn inhibition (Fig. 3J), suggesting that Fyn may amplify the inflammatory response through NF-κB pathway activation.

Collectively, these findings indicate that CD36-dependent proinflammatory polarization in BMDMs involves Fyn signaling. Fyn acts as a downstream effector that modulates cytokine production and NF-κB activation during M1 polarization.

### 3.6 CD36/Fyn Signaling Is Associated with CXCL Pathway Upregulation in M1 macrophages

**Fig. 4:**
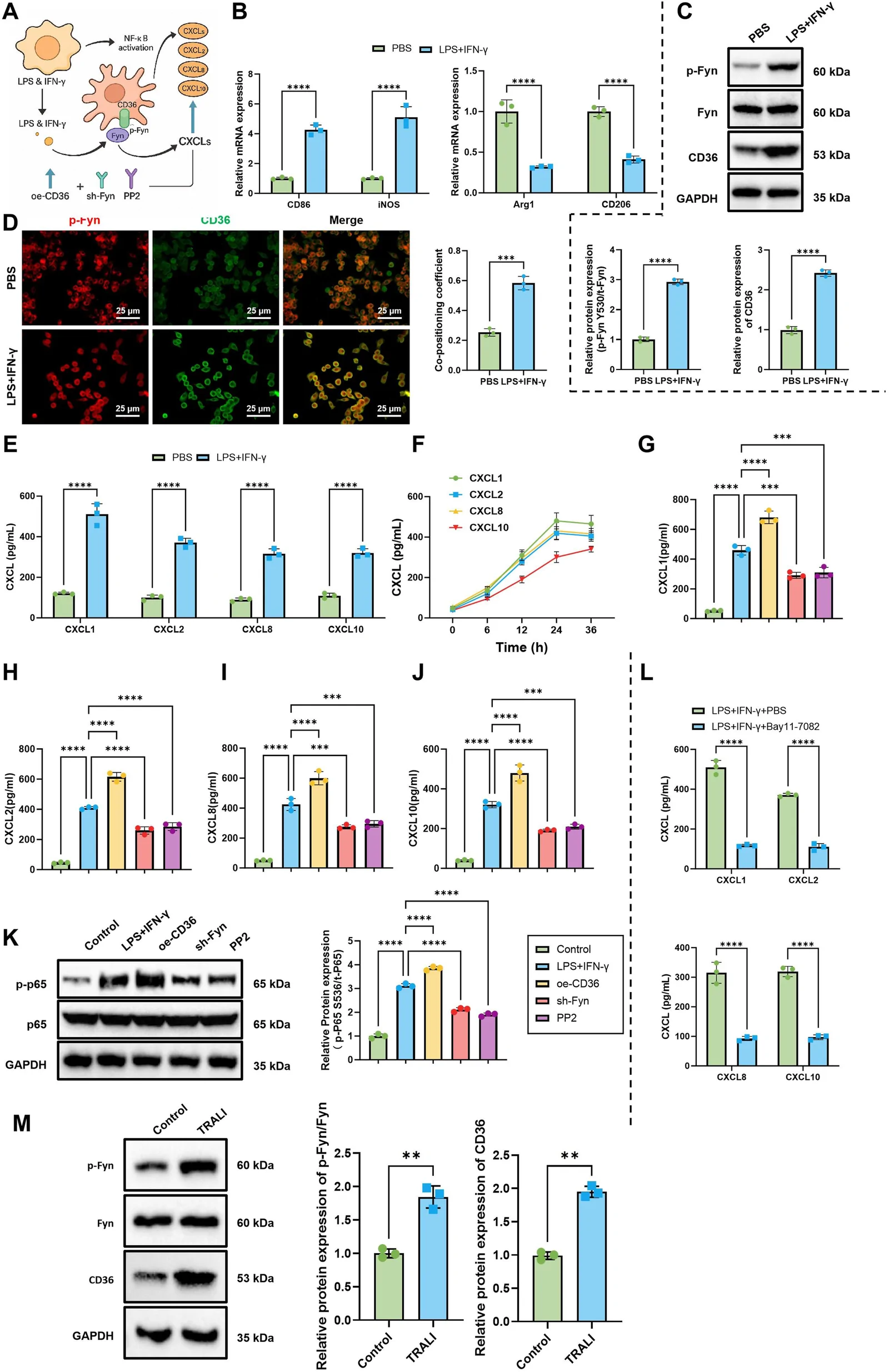
CD36/Fyn Signaling Is Associated with CXCL Pathway Upregulation in M1 Macrophages. Note: (A) Schematic illustration of the mechanism by which the CD36/Fyn signaling axis contributes to M1 macrophage polarization and promotes CXCL pathway activity; (B) RT-qPCR analysis of mRNA levels of pro-inflammatory markers (CD86, iNOS) and anti-inflammatory markers (Arg1, CD206) in M1-polarized macrophages; (C) Western blot analysis of CD36, phosphorylated Fyn (p-Fyn, Tyr530), and total Fyn protein levels in M1-polarized macrophages; (D) Immunofluorescence co-staining of CD36 (green) and p-Fyn (red) in macrophages, with nuclei counterstained by DAPI (blue); scale bar = 25 μm; (E) ELISA quantification of CXCL1, CXCL2, CXCL8, and CXCL10 secretion in the supernatants of M1-polarized macrophages; (F) Time-course ELISA analysis of CXCL1, CXCL2, CXCL8, and CXCL10 levels in macrophage supernatants at 0, 6, 12, 24, and 36 h after M1 polarization; (G-J) ELISA detection of CXCL1, CXCL2, CXCL8, and CXCL10 secretion following oe-CD36, sh-Fyn, or treatment with the Src family kinase inhibitor PP2; (K) Western blot analysis of NF-κB pathway activation via detection of p-p65 and total p65 after oe-CD36, sh-Fyn, or PP2 treatment; (L) ELISA assessment of CXCL1, CXCL2, CXCL8, and CXCL10 secretion following treatment with the NF-κB inhibitor Bay11-7082 in M1-polarized macrophages; (M) Western blot analysis of CD36, p-Fyn (Tyr530), and total Fyn protein levels in lung macrophages isolated from TRALI mice. Quantitative cell-based data in panels B-L were obtained from three independent biological experiments (*n* = 3 per group), with each plotted value representing one independent experiment. For panel M, lung macrophages were isolated independently from three biologically independent mice per group. ***p < 0.001; ****p < 0.0001.

To elucidate the role of CD36/Fyn signaling in M1 macrophage polarization and inflammatory chemokine regulation, we conducted a comprehensive analysis of macrophages stimulated with LPS and IFN-γ to induce M1 polarization (Fig. 4A). RT-qPCR results revealed a marked upregulation of proinflammatory markers CD86 and iNOS, alongside a significant reduction in anti-inflammatory markers Arg1 and CD206, confirming successful polarization toward the M1 phenotype (Fig. 4B). Based on this, Western blot analysis demonstrated elevated CD36 protein expression in M1 macrophages, accompanied by increased phosphorylation at Fyn Tyr530, while total Fyn levels remained unchanged (Fig. 4C). Dual immunofluorescence staining further showed that CD36 predominantly localized to the plasma membrane, whereas p-Fyn signals were concentrated in the submembranous and perinuclear regions. Notably, both signals exhibited strong colocalization at the membrane, supporting altered CD36-associated Fyn signaling under M1-polarized conditions (Fig. 4D). Subsequently, we assessed the secretion profile of CXCL chemokines. Because previous scRNA-seq screening indicated upregulation of CXCL1/2/8/10, these chemokines were selected as the focus of the subsequent analysis. ELISA results indicated that levels of CXCL1, CXCL2, CXCL8, and CXCL10 were significantly elevated in the supernatants of M1-polarized macrophages (Fig. 4E). A time-course experiment further revealed distinct temporal patterns: CXCL1 and CXCL2 exhibited rapid early-phase induction with peak expression at mid-phase, whereas CXCL10 showed a delayed yet sustained increase (Fig. 4F), indicating a dynamic, stage-specific activation of the CXCL signaling pathway.

To verify the signaling dependence of this process, we established an oe-CD36 system and a sh-Fyn system, supplemented with the Src family kinase inhibitor PP2. The results showed that oe-CD36 markedly enhanced the secretion of CXCL1/2/8/10, whereas both sh-Fyn and PP2 treatment significantly reduced CXCL levels (Fig. 4G–J). Concurrently, analysis of NF-κB signaling revealed that oe-CD36 elevated p-p65 levels, while inhibition of Fyn markedly attenuated this activation trend (Fig. 4K), suggesting that the CD36/Fyn axis may regulate CXCL transcription via NF-κB signaling. To further confirm NF-κB dependence, we examined CXCL secretion after blocking the NF-κB pathway with Bay11-7082, a commonly used NF-κB pathway inhibitor that irreversibly inhibits TNF-α-induced IκBα phosphorylation and degradation, prevents NF-κB nuclear translocation, and thereby suppresses target-gene transcription [21]. As an upstream regulatory molecule of NF-κB signaling, IκBα binds NF-κB p65 and maintains pathway inactivity, and its phosphorylation/degradation is a key step in NF-κB activation. The results demonstrated that NF-κB inhibition substantially decreased the expression levels of CXCL1/2/8/10 (Fig. 4L), indicating that upregulation of the CXCL family is transcriptionally regulated by NF-κB signaling. Lung macrophages were subsequently isolated from TRALI mice, and the results were similar to those observed in M1-polarized macrophages, further supporting the *in vivo* involvement of CD36/Fyn signaling (Fig. 4M).

Collectively, these findings indicate that M1 macrophage polarization is accompanied by altered CD36/Fyn signaling. The perturbation experiments support a role for Fyn in NF-κB pathway activity and CXCL chemokine production, providing a signaling basis for subsequent neutrophil recruitment and inflammatory amplification.

### 3.7 The CD36/Fyn-CXCL signaling axis drives neutrophil NET formation and amplifies inflammatory responses

**Fig. 5:**
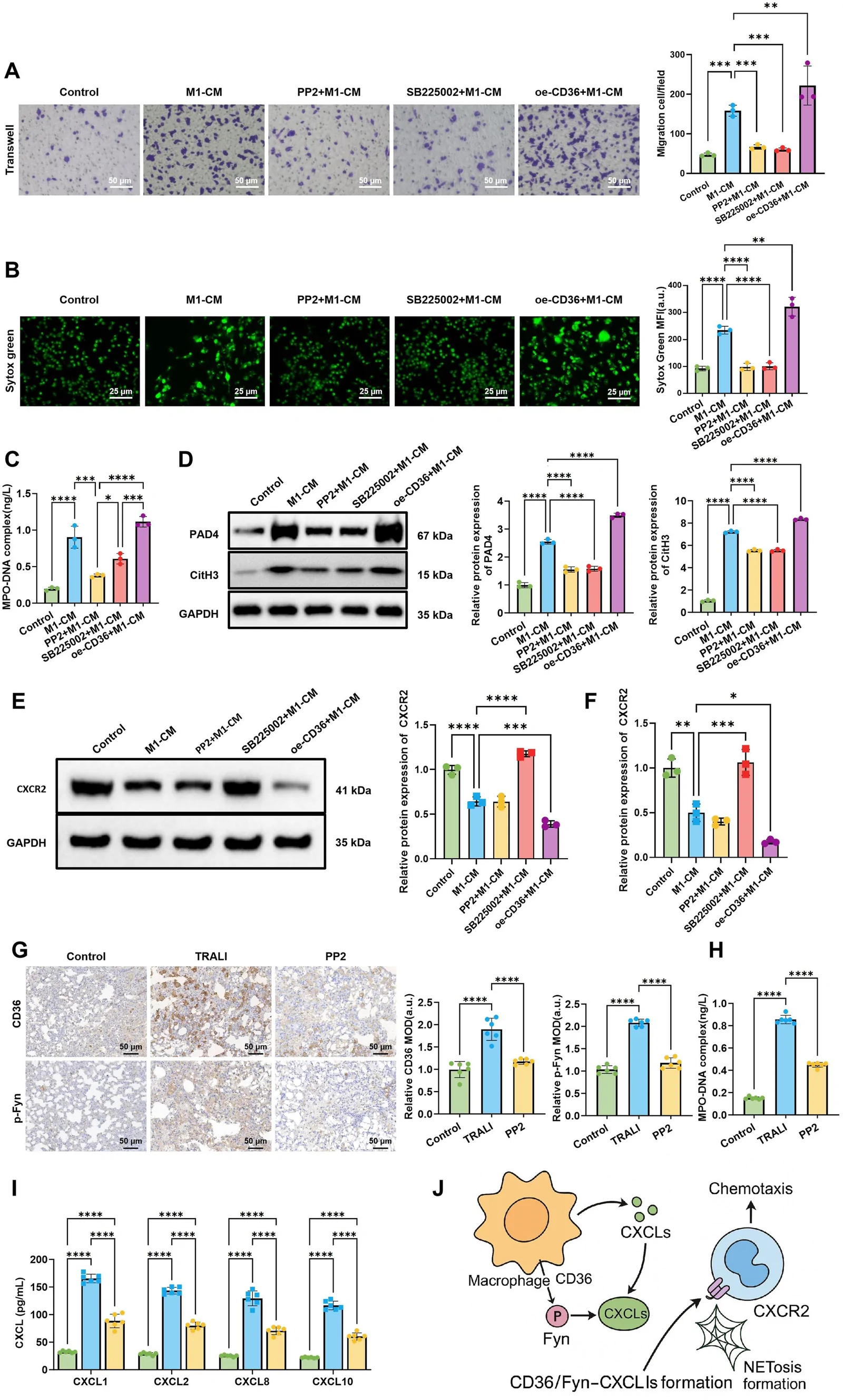
The CD36/Fyn-CXCL Signaling Pathway Drives Neutrophil Recruitment and NETosis Formation to Amplify Inflammation. Note: (A) Transwell migration assay assessing neutrophil migratory capacity under different treatment conditions; scale bar = 50 μm; (B) Sytox Green staining for detection of extracellular DNA/NETs (green) in neutrophils, with nuclear counterstaining by DAPI (blue); scale bar = 25 μm; (C) ELISA quantification of MPO-DNA complex levels in culture supernatants under various treatment conditions; (D) Western blot analysis of PAD4 and CitH3 protein expression in neutrophils; (E) Western blot analysis of CXCR2 protein expression in neutrophils; (F) qPCR analysis of CXCR2 mRNA expression in neutrophils; (G) IHC staining of CD36 and p-Fyn (Tyr530) proteins in lung tissues of TRALI model mice; scale bar = 50 μm; (H) ELISA quantification of MPO-DNA complexes in lung tissues; (I) ELISA analysis of CXCL1, CXCL2, CXCL8, and CXCL10 secretion in lung homogenates of TRALI model mice; (J) Schematic illustration of the role of the CD36/Fyn-CXCL signaling axis in mediating neutrophil chemotaxis and NETosis formation. Quantitative cell-based data in panels A-F were obtained from three independent biological experiments (*n* = 3 per group), with each plotted value representing one independent experiment. For the *in vivo* analyses in panels G-I, each group included six biologically independent mice. *p < 0.05; **p < 0.01; ***p < 0.001; ****p < 0.0001.

To investigate the role of the CD36/Fyn-CXCL signaling axis in neutrophil chemotaxis and NETosis, we established a co-culture system using M1-CM and neutrophils, and assessed neutrophil migration and NET formation under various intervention conditions. Transwell migration assays revealed that, compared with the Control group, neutrophil migration was significantly increased in the M1-CM group. In contrast, treatment with the Fyn inhibitor PP2 or the CXCR2 antagonist SB225002 markedly suppressed migration compared to the M1-CM group. Notably, neutrophil migration was partially restored in the oe-CD36 group despite PP2 treatment (Fig. 5A), indicating that CD36/Fyn-dependent signaling promotes neutrophil chemotaxis via the CXCL-CXCR2 pathway.

To further evaluate neutrophil NETosis, Sytox Green fluorescence staining was employed to detect extracellular DNA release. The results showed that, relative to the Control group, neutrophils in the M1-CM group exhibited abundant filamentous extracellular DNA structures with evident nuclear dispersion. Compared with the M1-CM group, PP2 and SB225002 treatments significantly reduced extracellular DNA release, whereas NET formation was markedly enhanced in the oe-CD36 group (Fig. 5B), suggesting that CD36/Fyn signaling facilitates NETosis. ELISA analysis further demonstrated that levels of MPO-DNA complexes were significantly elevated in the M1-CM group and were markedly reduced following treatment with PP2 or SB225002. In contrast, MPO-DNA complex levels in the oe-CD36 group rebounded significantly compared with the SB225002 group (Fig. 5C). In addition, Western blot analysis revealed that the protein levels of PAD4 and CitH3 in neutrophils were significantly upregulated in the M1-CM group compared with the Control group. These levels were notably downregulated in the PP2 and SB225002 groups but partially restored in the oe-CD36 group under Fyn inhibition (Fig. 5D). To determine whether the CD36/Fyn-CXCL axis affected CXCR2 receptor expression in neutrophils, CXCR2 protein and mRNA levels were detected by Western blot and qRT-PCR, respectively. Control neutrophils expressed relatively high levels of CXCR2, whereas M1-CM treatment markedly decreased CXCR2 expression, suggesting ligand-induced receptor internalization and downregulation driven by abundant CXCL chemokines. CXCR2 expression in the PP2 + M1-CM group was not statistically different from that in the M1-CM group, whereas SB225002 + M1-CM restored CXCR2 protein and mRNA levels to near, or slightly above, Control levels. Treatment with oe-CD36 macrophage-conditioned medium, which contained the highest CXCL levels, resulted in the lowest CXCR2 expression among the groups (Fig. 5E and F).

Further validation in lung tissues from the TRALI model mice revealed that, compared with the Control group, IHC showed markedly enhanced CD36 and p-Fyn (Tyr530) positive signals in the TRALI group. Following treatment with the Fyn inhibitor PP2, the expression levels of CD36 and p-Fyn were significantly reduced (Fig. 5G). In addition, ELISA results demonstrated that levels of MPO-DNA complexes, as well as CXCL1, CXCL2, CXCL8, and CXCL10, were significantly elevated in the TRALI group, whereas these indices were markedly decreased after PP2 intervention (Fig. 5H and I).

To further validate neutrophil NETosis responses driven by CXCL signaling, Sytox Green fluorescence staining was used to detect extracellular DNA release. Compared with the Control group, neutrophils in the CXCL2 group displayed abundant filamentous extracellular DNA structures with evident nuclear spreading. Compared with the CXCL2 group, SB225002 treatment significantly reduced extracellular DNA release, indicating that CXCL2 signaling promoted NETosis formation (Fig. S4).

Collectively, these findings indicate that M1-CM contains multiple NETosis-inducing mediators, including but not limited to CXCL, TNF-α, and IL-1β. CXCR2 inhibition partially reduced NETosis, suggesting that this axis participates in the process but is not the only pathway involved. NETosis levels were positively associated with the severity of lung injury, supporting its involvement in TRALI pathology. The CD36/Fyn signaling axis promotes CXCL-CXCR2 signaling, thereby enhancing neutrophil recruitment, and induces NETosis via PAD4-mediated histone citrullination, thereby amplifying the inflammatory cascade and contributing to tissue injury in TRALI (Fig. 5J).

### 3.8 EGCG suppresses the CD36/Fyn-CXCL signaling pathway to Modulate Macrophage Polarization and reduce NETosis formation

**Fig. 6:**
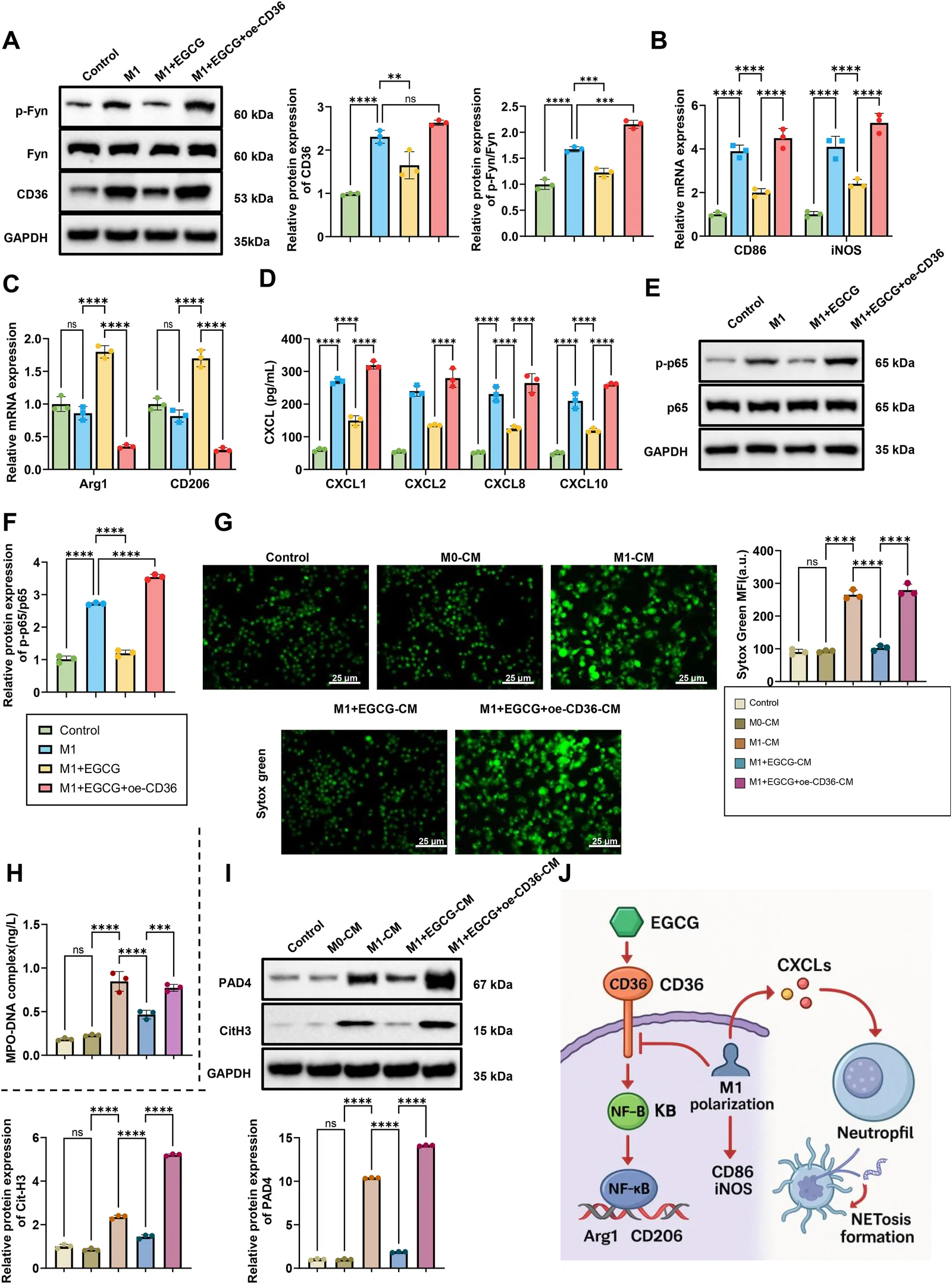
EGCG Inhibits the CD36/Fyn-CXCL Pathway to Modulate Macrophage Polarization and Attenuate NETosis Formation. Note: (A) Western blot analysis of CD36, p-Fyn (Tyr530), and total Fyn protein expression in macrophages; (B-C) RT-qPCR quantification of mRNA expression levels of pro-inflammatory markers (CD86, iNOS) and anti-inflammatory markers (Arg1, CD206) in macrophages under different treatment conditions; (D) ELISA measurement of CXCL1, CXCL2, CXCL8, and CXCL10 secretion in macrophage culture supernatants across treatment groups; (E-F) Western blot detection of NF-κB pathway-related proteins, including p-p65 and total p65, in treated macrophages; (G) Sytox Green staining to assess extracellular DNA/NETs formation (green) in neutrophils exposed to Control medium, M0-CM, M1-CM, M1 + EGCG-CM, or M1 + EGCG + oe-CD36-CM; nuclei were stained with DAPI (blue); scale bar = 25 μm; (H) ELISA quantification of MPO-DNA complex levels in neutrophil culture supernatants under various conditions; (I) Western blot analysis of PAD4 and CitH3 expression in neutrophils; (J) Schematic diagram illustrating the mechanism by which EGCG inhibits CD36/Fyn-CXCL-NF-κB signaling, suppresses M1 macrophage polarization, and reduces neutrophil NETosis. Quantitative cell-based data in panels A-I were obtained from three independent biological experiments (*n* = 3 per group), with each plotted value representing one independent experiment. ns indicates *p* ≥ 0.05; *p < 0.05; **p < 0.01; ***p < 0.001; ****p < 0.0001.

To investigate the regulatory effects of EGCG on the CD36/Fyn-CXCL signaling axis and its impact on macrophage polarization and neutrophil NETosis, we established an LPS + IFN-γ-induced M1 polarization system and designed four experimental groups for *in vitro* analysis: Control, M1, M1 + EGCG, and M1 + EGCG + oe-CD36. Western blot analysis showed that, compared with the Control group, CD36 and p-Fyn (Tyr530) levels were markedly increased in M1 macrophages; relative to the M1 group, EGCG treatment significantly downregulated both proteins. oe-CD36 restored their expression in the presence of EGCG, indicating that EGCG modulates CD36-associated Fyn phosphorylation and downstream macrophage signaling (Fig. 6A). Consistently, RT-qPCR analysis revealed that, compared with the Control group, the M1 group displayed substantial upregulation of pro-inflammatory markers CD86 and iNOS and downregulation of anti-inflammatory markers Arg1 and CD206. EGCG reversed these changes, whereas oe-CD36 partially reinstated the M1 phenotype (Fig. 6B and C).

ELISA results demonstrated that, compared with the Control group, CXCL1, CXCL2, CXCL8, and CXCL10 levels were markedly elevated in the supernatants of M1 macrophages. These chemokines were significantly reduced following EGCG treatment, while the M1 + EGCG + oe-CD36 group showed restored chemokine levels relative to EGCG alone, indicating that EGCG suppresses CXCL secretion by modulating CD36/Fyn-associated NF-κB signaling (Fig. 6D). Moreover, Western blot analysis showed that p-p65 was markedly increased in the M1 group but was substantially reduced after EGCG treatment, whereas p-p65 levels were increased again in the EGCG + oe-CD36 group, suggesting that EGCG inhibits inflammatory transcriptional activity by suppressing NF-κB activation (Fig. 6E and F).

To evaluate the regulatory effect of EGCG on NETosis, neutrophils were stimulated with macrophage-CM collected under different treatment conditions. NETosis and extracellular DNA release were assessed using Sytox Green staining (Fig. 6G). Compared with the Control group, the M1-CM group exhibited a substantial increase in green filamentous fluorescence, indicating enhanced extracellular DNA release. In contrast, EGCG treatment markedly reduced extracellular DNA release and attenuated the filamentous NET structures. However, oe-CD36 in the presence of EGCG restored extracellular DNA fluorescence and NET morphology. ELISA analysis further demonstrated that MPO-DNA complex levels were significantly elevated in the M1-CM group, markedly reduced following EGCG treatment, and subsequently increased again in the EGCG + oe-CD36 group (Fig. 6H). Western blot analysis revealed that PAD4 and CitH3 protein levels were significantly upregulated in the M1-CM group relative to the Control, indicating enhanced activation of NETosis-related enzymes. EGCG treatment suppressed the expression of both proteins, whereas oe-CD36 on top of EGCG treatment (M1 + EGCG + oe-CD36 group) restored their levels (Fig. 6I).

Collectively, in this *in vitro* overexpression model, EGCG treatment reduced CD36 and p-Fyn (Tyr530) levels in M1-polarized macrophages, suppressed NF-κB activation and CXCL chemokine secretion, and reduced neutrophil NETosis. It should be noted that EGCG is a natural polyphenol with broad antioxidant, anti-inflammatory, and kinase-regulatory activities, and the present study did not demonstrate direct binding to or specific inhibition of CD36. However, CD36 overexpression partially reversed these inhibitory effects, suggesting that downregulation of CD36-related signaling is an important component of the anti-inflammatory action of EGCG. EGCG may therefore serve as a tool compound for studying CD36/Fyn-related immune-inflammatory pathways, whereas its direct molecular binding mechanism requires further validation (Fig. 6J).

### 3.9 Fabrication and Characterization of a ROS-responsive PEV@SP@EG

**Fig. 7:**
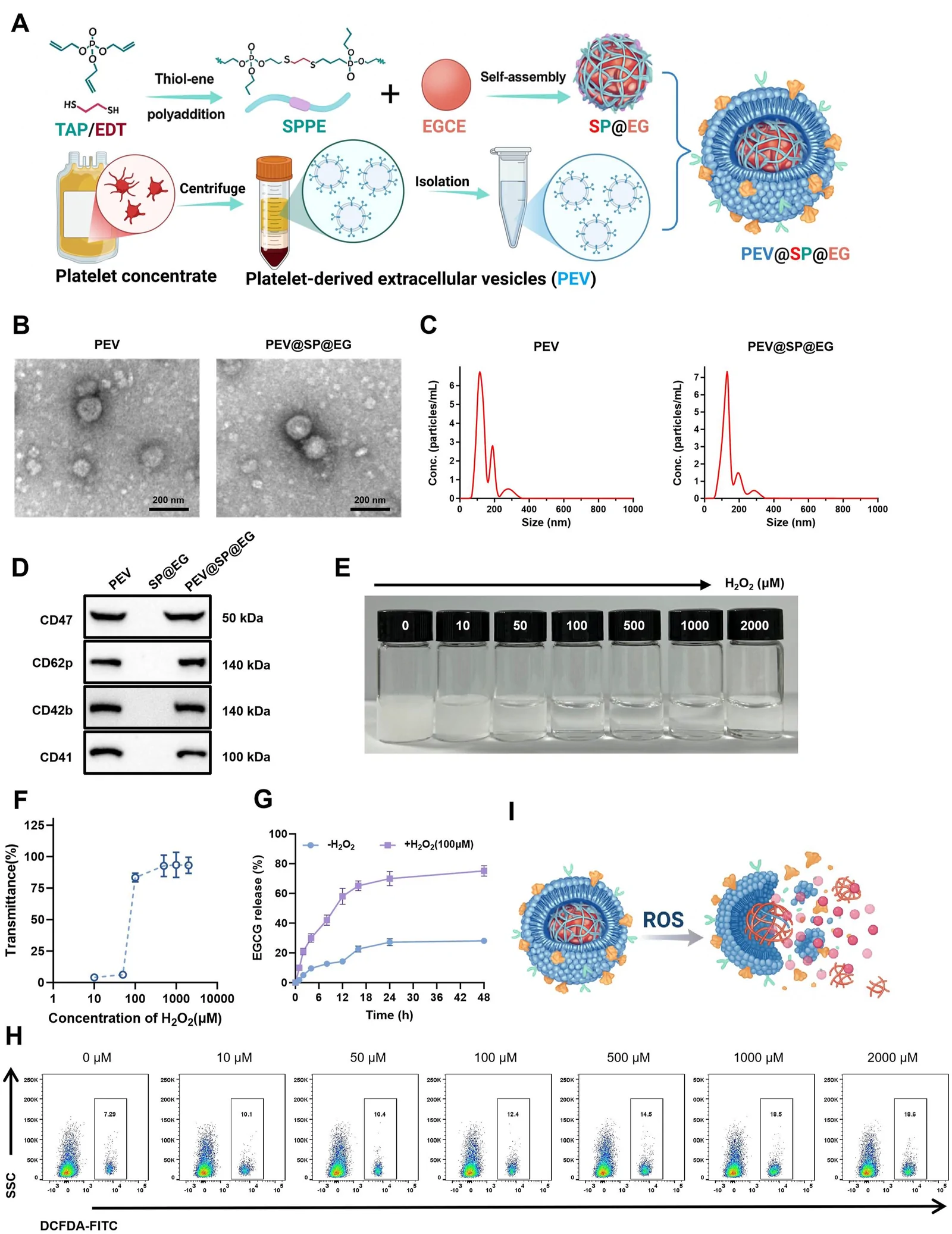
Fabrication and Characterization of the ROS-Responsive PEV@SP@EG. Note: (A) Schematic illustration of the preparation process of the PEV@SP@EG nanosystem, including the synthesis of the SPPE polymer, self-assembly of SP@EG, and coating with PEV; (B) TEM images showing the morphology of PEV and PEV@SP@EG; scale bar = 200 nm; (C) NTA of size distribution and concentration of PEV and PEV@SP@EG; (D) Western blot analysis of typical platelet membrane proteins (CD47, CD62p, CD42b, and CD41) in PEV and PEV@SP@EG; (E) Visual color changes of PEV@SP@EG solutions under varying H_2_O_2_ concentrations, indicating ROS responsiveness; (F) Measurement of transmittance of PEV@SP@EG solutions under different H_2_O_2_ concentrations; (G) EGCG release profiles from PEV@SP@EG in response to varying H_2_O_2_ concentrations; (H) DCFH-DA fluorescence detection of ROS responsiveness under different H_2_O_2_ concentrations; (I) Schematic representation of the ROS-triggered disassembly of PEV@SP@EG and subsequent EGCG release. Quantitative measurements were obtained from three replicate measurements per condition.

Given that EGCG effectively regulates macrophage polarization and reduces NETosis formation *in vitro* by inhibiting the CD36/Fyn-NF-κB signaling axis, we further explored a nanodelivery strategy to enhance its *in vivo* targeting specificity and pharmacological stability. To this end, we developed a ROS-responsive PEV@SP@EG and conducted a comprehensive characterization of its physicochemical properties and drug release behavior. Initially, SPPE polymer was synthesized via a thiol-ene click reaction and subsequently self-assembled with SP@EG to form a core-shell structure. PEVs were then isolated via ultracentrifugation and fused with SP@EG to form the final composite nanoparticle PEV@SP@EG (Fig. 7A).

TEM revealed that both PEV and PEV@SP@EG exhibited a spherical morphology with uniform dispersion and intact surface structure (Fig. 7B). NTA showed that PEV@SP@EG had a slightly larger particle size than PEV, displayed a unimodal distribution, and maintained stable concentration, indicating successful encapsulation of extracellular vesicles into a composite nanosystem (Fig. 7C). Western blot analysis confirmed the presence of characteristic platelet membrane markers—CD47, CD62p, CD42b, and CD41—on both PEV and PEV@SP@EG (Fig. 7D), suggesting effective retention of platelet-derived membrane proteins during the coating process. These proteins potentially endow the system with immune evasion and targeted adhesion capabilities. Upon exposure to varying concentrations of H_2_O_2_, the color of the PEV@SP@EG solution changed from milky white to transparent (Fig. 7E), and its transmittance increased significantly with rising H_2_O_2_ levels (Fig. 7F), indicating favorable ROS responsiveness of the nanosystem. DCFH-DA fluorescence intensity also gradually increased under increasing H_2_O_2_ concentrations (Fig. 7H), further confirming the ROS-responsive behavior of the nanosystem. Further EGCG release assays demonstrated that the system remained stable in the absence of H_2_O_2_, while drug release was significantly accelerated under high H_2_O_2_ concentrations (Fig. 7G), supporting its ROS-triggered controlled release capability.

In conclusion, the fabricated PEV@SP@EG nanosystem exhibited uniform morphology, structural stability, and effective preservation of platelet membrane protein features, along with excellent ROS responsiveness and controlled EGCG release performance (Fig. 7I).

### 3.10 PEV@SP@EG shows low hemolytic activity, No overt short-term cytotoxicity, and cellular uptake *in vitro*

To evaluate the biosafety and cellular uptake behavior of the ROS-responsive PEV@SP@EG, a comprehensive *in vitro* assessment was conducted (Fig. S5A). First, a hemolysis assay was performed to assess its compatibility with red blood cells. Across the tested concentrations, hemolysis remained below 5%, indicating low hemolytic activity under the experimental conditions (Fig. S5B).

Next, Calcein-AM/PI dual staining was used to assess macrophage viability. Across the tested concentrations, cells predominantly exhibited green fluorescence, with minimal red PI staining and intact cellular morphology, and no overt reduction in the predominance of viable cells was observed (Fig. S5C). Annexin V/PI flow cytometry likewise showed no marked increase in the measured apoptotic-cell proportion relative to the control under the tested conditions (Fig. S5D). Together, these assays detected no overt short-term cytotoxic response, although the non-significant comparisons were not interpreted as evidence of equivalence between groups.

For the cellular uptake study, PEVs were labeled with green fluorescence, and EGCG was conjugated with red TAMRA, while nuclei were stained with blue DAPI. Confocal microscopy revealed partial co-localization of red and green signals within macrophages (Fig. S5E), indicating efficient internalization of PEV@SP@EG and successful intracellular delivery of the therapeutic cargo.

In summary, PEV@SP@EG showed low hemolytic activity, no overt short-term reduction in cell viability or marked increase in apoptosis under the tested conditions, and detectable cellular uptake *in vitro*.

### 3.11 The PEV@SP@EG nanosystem Exhibits favorable *in vivo* safety

To assess the *in vivo* safety of the ROS-responsive PEV@SP@EG, healthy mice were intravenously injected via the tail vein with PBS, SP@EG, or PEV@SP@EG. Before euthanasia, serum biochemical and histological analyses were performed (Fig. S6A).

H&E staining revealed intact tissue architecture in the major organs (heart, liver, spleen, lung, kidney) across all groups, with orderly cellular arrangement and no evidence of inflammation, necrosis, or hemorrhage. Compared with the PBS group, the PEV@SP@EG-treated group exhibited no notable morphological changes in organ tissues, indicating that the nanosystem did not elicit visible tissue toxicity at physiological doses (Fig. S6B). Serum biochemical analysis further showed that ALT and AST levels in the PEV@SP@EG group were comparable to those in the PBS group, indicating no hepatotoxicity. Similarly, BUN and CRE levels did not significantly differ from controls, indicating preserved renal function (Fig. S6C). Complete blood count analysis demonstrated that RBC, WBC, NEU, and HGB levels remained within normal ranges, with no significant differences observed among the PBS, SP@EG, and PEV@SP@EG groups (Fig. S6D). Additionally, ELISA results showed that serum levels of inflammatory cytokines IL-6, IFN-γ, and TNF-α were not significantly elevated in the PEV@SP@EG-treated mice, following a similar trend to the SP@EG group, suggesting that the nanosystem did not induce acute systemic inflammation (Fig. S6E).

Collectively, these findings indicate that the PEV@SP@EG nanosystem possesses excellent *in vivo* biocompatibility and safety, without causing major organ toxicity, hematological abnormalities, or systemic inflammatory responses at the experimental dosage.

### 3.12 *In vivo* targeting evaluation of the PEV@SP@EG nanosystem

To assess the biodistribution and pulmonary targeting capability of the ROS-responsive PEV@SP@EG, DiR-labeled SP@EG or PEV@SP@EG nanoparticles were intravenously administered via the tail vein in mice, followed by *in vivo* fluorescence imaging at various time points (Fig. S7A).

*In vivo* imaging results showed that in the SP@EG group, strong fluorescence signals initially appeared in the liver and spleen regions at 3 h post-injection but rapidly diminished and were nearly eliminated by 48 h. In contrast, the PEV@SP@EG group exhibited pronounced fluorescence accumulation in the lung region at 12 h post-injection, peaking at 24 h, with detectable signals persisting up to 48 h (Fig. S7B). These findings indicate that coating with PEVs markedly altered the nanosystem's *in vivo* biodistribution, enhancing its pulmonary targeting and accumulation.

*Ex vivo* fluorescence imaging of isolated organs further demonstrated that SP@EG was predominantly distributed in the liver and spleen, whereas PEV@SP@EG exhibited higher pulmonary accumulation; the *ex vivo* organ images also included TRALI model mice injected with PEV@SP@EG for lung-targeting assessment (Fig. S7C). Quantitative analysis confirmed that the fluorescence signal of PEV@SP@EG in lung tissue was significantly higher than that of the SP@EG group in the formulation comparison (Fig. S7D), and the corresponding organ biodistribution data are summarized in Table S3. Confocal microscopy further verified the microscale distribution of PEV@SP@EG in lung tissue. The results revealed that PEV@SP@EG nanoparticles (red fluorescence) were primarily localized in the alveolar septa and capillary endothelial regions, with distinct spatial correspondence to DAPI-stained nuclei (blue), suggesting effective lung-targeted delivery (Fig. S7E).

In summary, biomimetic modification with PEVs substantially improved the *in vivo* distribution profile of the nanosystem, endowing PEV@SP@EG with superior pulmonary targeting and enrichment capabilities.

### 3.13 PEV@SP@EG significantly alleviates lung injury and suppresses inflammatory responses in TRALI mice

**Fig. 8:**
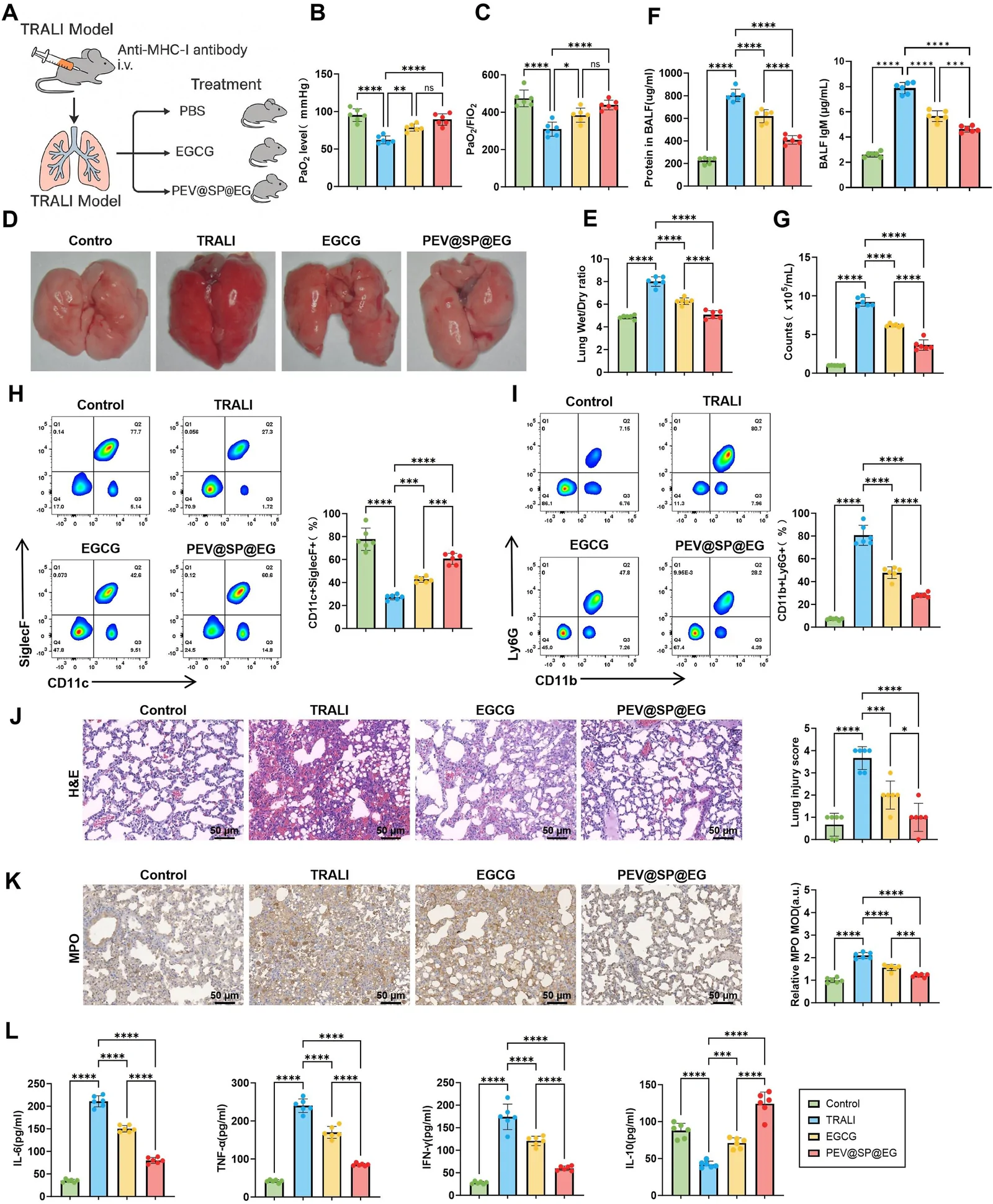
Therapeutic Efficacy of the PEV@SP@EG Nanosystem in TRALI Mice. Note: (A) Schematic diagram illustrating the TRALI model induction and treatment protocols for the PBS, EGCG, and PEV@SP@EG groups; (B-C) Blood gas analysis of PaO_2_ and PaO_2_/FiO_2_; (D) Representative gross images of lung tissues from each group; (E) Lung W/D to assess pulmonary edema; (F) TP and IgM content in BALF; (G) Total cell count in BALF; (H) Flow cytometric analysis of CD11c^+^SiglecF^+^ alveolar macrophage proportion in BALF; (I) Flow cytometric analysis of CD11b^+^Ly6G^+^ neutrophil proportion in BALF; (J) H&E staining of lung tissues to evaluate histological alterations; scale bar = 50 μm; (K) IHC staining of MPO to visualize positive signals in lung tissues; scale bar = 50 μm; (L) ELISA quantification of IL-6, TNF-α, IFN-γ, and IL-10 levels in lung homogenates and serum. Each group contained six animals. *p < 0.05, **p < 0.01, ***p < 0.001, ****p < 0.0001 compared between groups.

To evaluate the therapeutic potential of the PEV@SP@EG nanosystem in TRALI, a murine TRALI model was established by anti-MHC-I antibody induction, followed by intravenous administration of PBS, EGCG, or PEV@SP@EG, respectively (Fig. 8A).

Blood gas analysis revealed that the TRALI group exhibited a significant reduction in PaO_2_ and PaO_2_/FiO_2_, whereas treatment with EGCG or PEV@SP@EG markedly restored oxygenation capacity (Fig. 8B and C). Macroscopically, lung tissues from the TRALI group displayed pronounced congestion and edema, while the EGCG and nanosystem-treated groups demonstrated near-normal appearance (Fig. 8D).

Measurement of the lung W/D showed a significant increase in pulmonary edema in the TRALI group, which was effectively reduced following treatment with EGCG or PEV@SP@EG (Fig. 8E). Analysis of BALF further indicated that TP concentration, total cell count, and IgM levels were markedly elevated in the TRALI group but significantly decreased after EGCG or PEV@SP@EG administration (Fig. 8F and G), suggesting improved alveolar-capillary barrier integrity.

Flow cytometric analysis was further conducted to evaluate changes in the cellular composition of BALF. In the TRALI group, the proportion of CD11c^+^SiglecF^+^ alveolar macrophages was significantly reduced, whereas this population was restored to near-normal levels following PEV@SP@EG treatment (Fig. 8H). Conversely, the proportion of CD11b^+^Ly6G^+^ neutrophils was markedly elevated in the TRALI group but significantly decreased after intervention with EGCG or PEV@SP@EG (Fig. 8I). H&E staining of lung tissue revealed severe alveolar structural disruption and prominent inflammatory cell infiltration in the TRALI group, while structural integrity was notably improved in the PEV@SP@EG-treated group (Fig. 8J). IHC analysis showed a substantial increase in MPO-positive signals in the TRALI group, which was markedly reduced following PEV@SP@EG administration (Fig. 8K). ELISA was further employed to assess inflammatory cytokine levels. The results demonstrated elevated concentrations of IL-6, TNF-α, and IFN-γ in the TRALI group, all of which were significantly decreased after PEV@SP@EG treatment. In contrast, IL-10 levels were significantly increased in the PEV@SP@EG group (Fig. 8L). Total cell count, PMN absolute count, and PMN percentage in BALF were also increased in the model group and were reversed after PEV@SP@EG treatment (Table S2).

Taken together, these findings indicate that the PEV@SP@EG nanosystem effectively improves oxygenation, alleviates pulmonary edema, reduces inflammatory cell infiltration, and modulates cytokine profiles in TRALI mice.

### 3.14 PEV@SP@EG blocks the CD36/Fyn-NF-κB-CXCL axis to suppress inflammatory cascades and NETosis formation

**Fig. 9:**
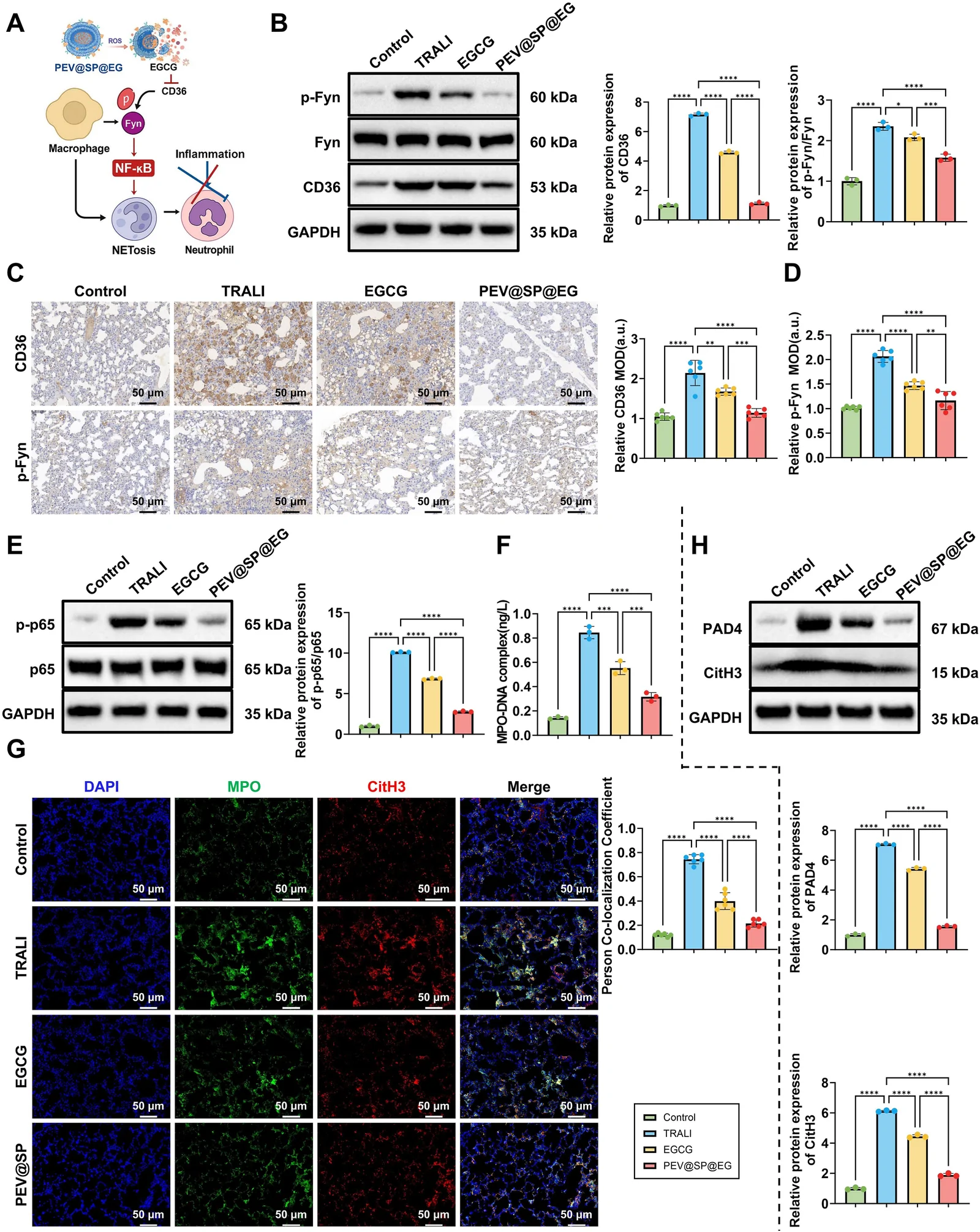
Analysis of CD36-Fyn Signaling, NETs Formation, and Inflammatory Cytokine Expression in Lung Tissues of TRALI Mice Treated with PEV@SP@EG Nanosystem. Note: (A) Schematic illustration of the proposed mechanism by which PEV@SP@EG releases EGCG to modulate CD36/Fyn-associated signaling, block the NF-κB-CXCL pathway, and consequently attenuate macrophage pro-inflammatory polarization and neutrophil NETosis formation; (B) Western blot analysis of CD36, p-Fyn (Tyr530), and total Fyn protein expression in lung tissues across treatment groups, along with densitometric quantification of CD36 expression and the p-Fyn (Tyr530)/Fyn ratio; (C-D) IHC staining of CD36 and p-Fyn (Tyr530) in lung tissues, with representative images and semi-quantitative analysis of MOD for CD36 and p-Fyn (Tyr530); scale bar = 50 μm; (E) Western blot analysis of the p-NF-κB p65/NF-κB p65 protein expression ratio in lung tissues; (F) ELISA quantification of MPO-DNA complex levels; (G) Immunofluorescence staining to assess NETs formation: DAPI for nuclei (blue), MPO for neutrophil granules (green), and CitH3 for NET-associated histones (red); white arrows indicate representative NETs-positive regions; scale bar = 50 μm; (H) Western blot analysis of PAD4 and CitH3 protein expression. All quantitative *in vivo* data shown in Fig. 9 were analyzed using six biologically independent mice per group (*n* = 6/group). Biologically independent animals were treated as the experimental units, and technical or assay-level repeated measurements were not treated as independent biological replicates. *p < 0.05, **p < 0.01, ***p < 0.001, ****p < 0.0001.

Cell-based experiments previously demonstrated that EGCG modulates CD36/Fyn-associated signaling, thereby inhibiting the NF-kappaB-CXCL pathway, reducing inflammatory cytokine secretion, and attenuating neutrophil NETosis. To verify whether the ROS-responsive release of EGCG from PEV@SP@EG *in vivo* exerts a similar regulatory mechanism (Fig. 9A), we assessed the expression levels of key signaling proteins associated with macrophage polarization. Western blot analysis revealed that CD36 and p-Fyn (Tyr530) levels were significantly elevated in the TRALI group, with no observable change in total Fyn. Treatment with EGCG or PEV@SP@EG significantly reduced both CD36 and p-Fyn (Tyr530) levels (Fig. 9B). IHC staining further confirmed this trend: strong positive signals for CD36 and p-Fyn (Tyr530) were observed in the alveolar walls and perivascular regions of the TRALI group, whereas these signals were markedly diminished following PEV@SP@EG treatment (Fig. 9C and D). Meanwhile, to further elucidate changes in downstream signaling pathways, we examined the activation status of the NF-kappaB pathway. The ratio of phosphorylated NF-kappaB p65 to total NF-kappaB p65 was significantly elevated in the TRALI group, whereas both EGCG and PEV@SP@EG treatments effectively reduced this ratio (Fig. 9E). Finally, NETs-related markers were examined. ELISA results showed a significant increase in MPO-DNA complex levels in the lung tissue of the TRALI group, which was notably reduced following PEV@SP@EG treatment (Fig. 9F). Immunofluorescence staining further revealed strong colocalization of CitH3 and MPO signals in TRALI lung tissues, indicating extensive NETs formation, whereas such colocalization was markedly diminished after PEV@SP@EG treatment (Fig. 9G). Western blot analysis showed that the expression of CitH3 and PAD4 proteins was significantly elevated in the TRALI group but was substantially downregulated after PEV@SP@EG intervention (Fig. 9H). Together, these findings indicate that the PEV@SP@EG nanosystem attenuates dysregulated CD36/Fyn-associated signaling and NETosis-related inflammatory responses in TRALI lung tissue. Statistical sensitivity analysis for the four-group *in vivo* design is shown in Fig. S8, with endpoint-specific results provided in Table S6. The corresponding design-specific sensitivity analysis for cell-based quantitative measurements is shown in Fig. S9, with measurement-specific results provided in Table S7.

**Fig. 10:**
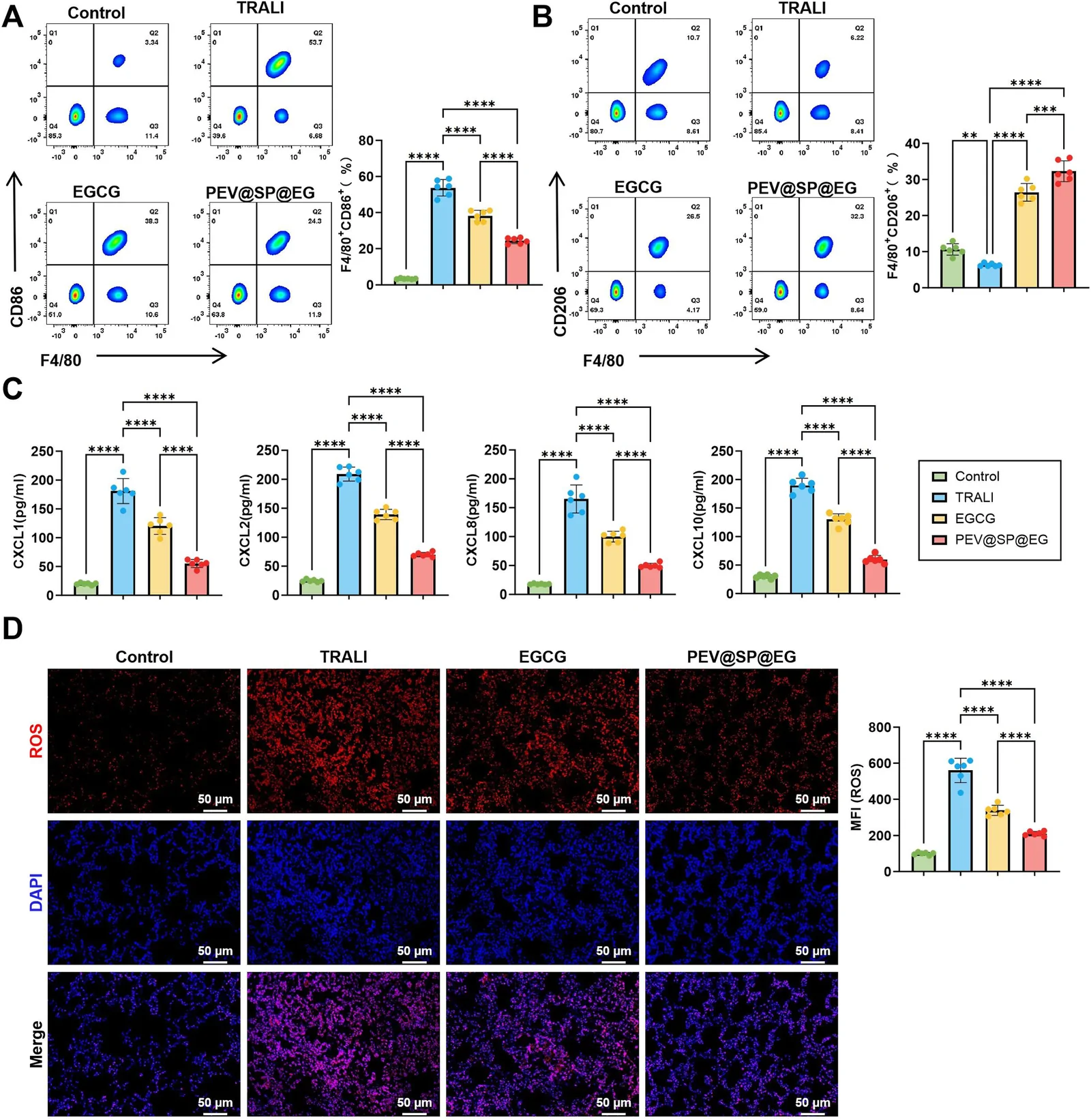
Regulatory Effects of PEV@SP@EG on Macrophage Polarization and CXCL-ROS Signaling. Note: (A-B) Flow cytometry analysis of the proportions of F4/80^+^CD86^+^ and F4/80^+^CD206^+^ macrophages in lung tissues; (C) ELISA quantification of CXCL1, CXCL2, CXCL8, and CXCL10 levels in lung tissue homogenates and BALF; (D) Detection of total ROS levels in lung tissues using a DCFH-DA probe; scale bar = 50 μm. Each group included six biologically independent mice. **p < 0.01, ***p < 0.001, ****p < 0.0001.

To evaluate the impact of the PEV@SP@EG nanosystem on macrophage polarization and downstream CXCL-ROS signaling in the lung tissue of TRALI mice, flow cytometry was employed to quantify the proportions of F4/80^+^CD86^+^ and F4/80^+^CD206^+^ cells. Compared to the PBS group, the TRALI group exhibited a significant increase in F4/80^+^CD86^+^ double-positive cells and a marked decrease in F4/80^+^CD206^+^ cells, indicating a shift toward the pro-inflammatory M1 phenotype under TRALI conditions. Following treatment with the PEV@SP@EG nanosystem, the proportion of F4/80^+^CD86^+^ cells was significantly reduced, whereas F4/80^+^CD206^+^ cells increased notably, suggesting that the system effectively inhibits M1 polarization and promotes restoration of the anti-inflammatory M2 phenotype (Fig. 10A and B). Moreover, ELISA analysis revealed that levels of CXCL1, CXCL2, CXCL8, and CXCL10 were markedly increased in lung homogenates and BALF of the TRALI group, while PEV@SP@EG treatment significantly downregulated the expression of these chemokines (Fig. 10C). Concurrently, DCFH-DA probe detection revealed a pronounced accumulation of total ROS in TRALI lung tissues, which was significantly attenuated by PEV@SP@EG treatment (Fig. 10D). Together, these findings demonstrate that PEV@SP@EG suppresses macrophage M1 polarization, reduces CXCL secretion, and mitigates ROS accumulation in TRALI lung tissue.

In summary, PEV@SP@EG modulated CD36/Fyn-associated signaling, NF-kappaB-mediated CXCL secretion, neutrophil NETosis, and oxidative stress, thereby supporting lung-protective effects in TRALI. These coordinated molecular and cellular changes provide additional mechanistic insight into ROS-responsive nanotherapeutic intervention in this model.

## 4 Discussion

TRALI is a severe transfusion complication characterized by rapid pulmonary inflammation, endothelial barrier disruption, and impaired gas exchange. Macrophages and neutrophils are recognized as key immune mediators in this process, but the signaling events that connect macrophage activation to neutrophil recruitment and NET formation remain incompletely understood. In this study, we combined exploratory single-cell transcriptomic analysis, *in vitro* mechanistic experiments, and an *in vivo* TRALI model to examine whether macrophage CD36/Fyn signaling contributes to CXCL-dependent neutrophil activation and NETosis. We further evaluated a ROS-responsive biomimetic nanoplatform, PEV@SP@EG, designed to deliver EGCG to injured lung tissue and suppress this inflammatory cascade.

Single-cell analysis of lung tissue from the TRALI model indicated broad immune remodeling and identified macrophages as a major compartment associated with inflammatory activation. CD36 expression was enriched in macrophage populations with M1-like features, suggesting a potential role for CD36 in shaping the inflammatory state of macrophages during TRALI. Although these single-cell data were exploratory, they provided a rationale for subsequent functional validation. In BMDMs, CD36 overexpression increased M1 polarization markers, CXCL production, Fyn-associated phosphorylation changes and NF-kappaB activation, and ROS generation, whereas CD36 knockdown or EGCG treatment reversed these effects. These findings support the view that CD36 acts upstream of Fyn/NF-kappaB signaling in macrophage inflammatory activation.

The macrophage-neutrophil interaction was further supported by conditioned-medium experiments. Medium from CD36-overexpressing macrophages promoted neutrophil migration and NETosis, while CXCL-CXCR2 blockade reduced these responses. These results suggest that CXCL signaling contributes to the ability of activated macrophages to recruit and stimulate neutrophils. Because NETosis can intensify endothelial injury and inflammatory amplification in TRALI, interruption of this macrophage-derived chemokine signal may represent a useful therapeutic direction. The observation that CD36/Fyn inhibition weakened both chemokine production and NET formation links upstream macrophage signaling to downstream neutrophil effector activity.

A major challenge in treating TRALI is the need to deliver anti-inflammatory agents to the injured lung while limiting systemic exposure. PEV@SP@EG was designed to address this issue by combining platelet extracellular vesicle-mediated pulmonary targeting with ROS-responsive EGCG release. *In vitro* characterization showed that PEV@SP@EG maintained vesicle-like morphology and released EGCG more efficiently under oxidative conditions. *In vivo* imaging demonstrated preferential lung accumulation, including in TRALI model mice, supporting the feasibility of this biomimetic delivery strategy in an inflammatory pulmonary microenvironment.

Therapeutically, PEV@SP@EG alleviated lung injury in the TRALI model, as shown by improved histopathological appearance, reduced edema and permeability indices, decreased inflammatory cytokine levels, and improved blood gas parameters. Mechanistically, treatment suppressed CD36/Fyn-NF-kappaB signaling, reduced CXCL expression, limited neutrophil infiltration and NET formation, and attenuated oxidative stress. These effects were consistent with the *in vitro* findings and suggest that the nanoplatform acts on both macrophage inflammatory activation and neutrophil-mediated tissue injury.

Compared with free EGCG, PEV@SP@EG may offer advantages related to targeted delivery and ROS-responsive release. EGCG has recognized anti-inflammatory and antioxidant properties, but its therapeutic application can be constrained by limited stability and bioavailability. Encapsulation within a platelet extracellular vesicle-based system may improve delivery to inflamed lung tissue and increase the local availability of EGCG in an oxidative environment. This strategy therefore provides a potential means to enhance the pharmacological utility of EGCG in acute inflammatory lung injury.

Several limitations should be acknowledged. First, this work focused on CD36/Fyn signaling in M1-like macrophage activation, and the characteristics of untreated or M0 macrophages were not extensively profiled. Second, only male mice were used; therefore, potential sex-related differences in TRALI responses and therapeutic efficacy remain a limitation, and future studies should include both sexes. Third, the single-cell transcriptomic analysis was based on one mouse per group and should be interpreted as exploratory; larger cohorts are needed for validation. Fourth, most cell-based mechanistic assays were performed in three independent biological experiments. Although the major mechanistic trends were reproducible across these experiments, additional independent experiments would improve the precision of effect-size estimates and strengthen the generalizability of the findings. Statistical sensitivity varied among quantitative measurements, particularly for small or null effects. Fifth, the bioavailability, pharmacokinetics, and long-term safety of PEV@SP@EG require further investigation. Sixth, additional TRALI models and mouse strains will be needed to evaluate generalizability.

In conclusion, this study identifies a CD36/Fyn-NF-kappaB-CXCL axis that links macrophage activation with neutrophil recruitment and NETosis in TRALI. The ROS-responsive platelet extracellular vesicle biomimetic nanosystem PEV@SP@EG attenuated this inflammatory cascade and improved lung injury outcomes in the experimental model. These findings provide mechanistic support for targeting macrophage-neutrophil crosstalk in TRALI and highlight PEV-based ROS-responsive delivery as a promising platform for future therapeutic development.

## CRediT authorship contribution statement

**Jiani Gao:** Conceptualization. **Ke Zhou:** Conceptualization. **Yijiu Ren:** Conceptualization. **Fengjing Yang:** Conceptualization. **Minjue Shan:** Conceptualization. **Yubing Fang:** Conceptualization. **Changbo Sun:** Conceptualization. **Weili Han:** Conceptualization. **Chang Chen:** Funding acquisition, Conceptualization. **Xuefei Hu:** Writing – review & editing, Supervision, Funding acquisition, Conceptualization.

## Ethics declaration

This study was conducted in accordance with the ARRIVE (Animal Research: Reporting of In Vivo Experiments) guidelines. This study was approved by the Shanghai Pulmonary Hospital Medical Ethics Committee. (Approval No. K24-640Z)

## Funding

This work was supported by 10.13039/501100001809Noncommunicable Chronic Diseases of National Science and Technology Major Project (2024ZD0529000, ), 10.13039/100018925National Natural Science Foundation of China (82302004, 82606720), and Zhejiang Provincial Natural Science Foundation of China LQ24H100003.

## Declaration of competing interests

The authors declare that they have no known competin_g_ financial interests or personal relationships that could have appeared to influence the work reported in this paper.

## Data Availability

Data will be made available on request.

## Abbreviations

ALT: Alanine Aminotransferase
AST: Aspartate Aminotransferase
BALF: Bronchoalveolar Lavage Fluid
BMDMs: Bone Marrow-Derived Macrophages
BP: Biological Process
BUN: Blood Urea Nitrogen
CC: Cellular Component
CitH3: Citrullinated Histone H3
CM: Conditioned Medium
CRE: Creatinine
DEGs: Differentially Expressed Genes
EGCG: Epigallocatechin Gallate
ELISA: Enzyme-Linked Immunosorbent Assay
FBS: Fetal Bovine Serum
GO: Gene Ontology
H&E: Hematoxylin and Eosin
H_2_O_2_: Hydrogen Peroxide
IHC: Immunohistochemistry
KEGG: Kyoto Encyclopedia of Genes and Genomes
LPS: Lipopolysaccharide
M-CSF: Macrophage Colony-Stimulating Factor
MF: Molecular Function
MOD: Mean Optical Density
MPO: Myeloperoxidase
NETs: Neutrophil Extracellular Traps
NETosis: Neutrophil Extracellular Trap-osis
NK: Natural Killer
NTA: Nanoparticle Tracking Analysis
OD: Optical Density
oe-CD36: CD36 Overexpression
PCA: Principal Component Analysis
PCs: Principal Components
PBS: Phosphate-Buffered Saline
PEV: Platelet-Derived Extracellular Vesicle
PEV@SP@EG: Platelet-Derived Extracellular Vesicle-Coated ROS-Responsive Sulfur-Containing Poly(Phosphate Ester) Nanoparticles Loaded with Epigallocatechin Gallate
ROS: Reactive Oxygen Species
scRNA-seq: Single-Cell RNA Sequencing
sh-CD36: CD36 Knockdown
SP@EG: Epigallocatechin Gallate-Loaded Sulfur-Containing Poly(Phosphate Ester) Nanoparticles
SPPE: Sulfur-Containing Poly(Phosphate Ester)
TEM: Transmission Electron Microscopy
TP: Total Protein
TRALI: Transfusion-Related Acute Lung Injury
W/D: Wet/Dry Weight Ratio

## Appendix A Supplementary data

Multimedia component 2

Fig S1 Histological and Inflammatory Characteristics of Lung Tissue in TRALI Model Mice. Note: (A) H&E staining showing structural changes in lung tissue from Control and TRALI groups; bar = 50 μm; (B) Pathological lung injury score; (C) TP concentration in BALF; (D) Lung W/D; (E) IHC localization of epithelial tight junction proteins ZO-1 and E-cadherin in lung tissue; bar = 50 μm; (F) Quantitative analysis of ZO-1 and E-cadherin IHC signal intensities; (G) ELISA analysis of inflammatory cytokines TNF-α, IL-1β, IL-6, and IL-10 levels in BALF; (H) Masson's trichrome staining; (I-J) blood gas analysis of arterial PaO_2_ and PaO_2_/FiO_2_ showing collagen fiber distribution in lung tissue; bar = 50 μm. Each group included six animals. *****p* < 0.0001.

Fig S2 Quality Control and Dimensionality Reduction of scRNA-seq Data. Note: (A) Scatter plots of cellular quality control metrics, showing relationships between total transcripts (nCount_RNA), detected genes (nFeature_RNA), percentage of mitochondrial genes (percent.mt), and percentage of hemoglobin-related genes (percent.HB) versus nCount_RNA; (B) PCA results: the left panel displays the PC1 vs. PC2 embedding plot, while the right panel shows violin plots of PC1 distribution across sample groups; (C) Heatmap of top-contributing genes for the first six PCs, representing highly weighted genes within each component.

Fig S3 CD36 Expression Regulates Macrophage Polarization Status. Note: (A) UMAP visualization showing the distribution pattern of *Cd36* expression across macrophage subclusters; (B) descriptive single-cell-level co-expression assessment between *Cd36* and representative M1-related genes, including Cd86, Ccl11, Il1b, Ccl3, Ccl5, Stat1, Tnf, and Nfkb1. (C) schematic illustration of CD36 overexpression and knockdown in macrophages; (D) Western blotting and densitometric analysis of iNOS and CD86 in the oe-NC, oe-CD36, sh-NC, and sh-CD36 groups; (E) flow cytometric analysis and quantification of CD86-positive macrophages; (F) ELISA quantification of IL-1β, TNF-α, IL-6, and IL-18 in culture supernatants; (G) DCFH-DA fluorescence imaging and quantitative analysis of intracellular ROS; scale bar = 25 μm. The single-cell analyses in panels A-B were exploratory and were not interpreted as sample-level statistical inference. Quantitative cell-based data in panels D-G were obtained from three independent biological experiments (*n* = 3 per group), with each plotted value representing one independent experiment. **p* < 0.05; ***p* < 0.01; ****p* < 0.001; *****p* < 0.0001.

Fig S4 CXCL Signaling Drives Neutrophil Recruitment and NETosis Formation. Note: Sytox Green staining was used to visualize extracellular DNA/NETs formation in neutrophils exposed to Control medium, CXCL2, or CXCL2 plus SB225002; nuclei were counterstained with DAPI (blue); scale bar = 25 μm. Representative images from three independent biological experiments are shown.

Fig S5 Biocompatibility and Cellular Uptake Evaluation of PEV@SP@EG. Note: (A) Schematic diagram of the experimental design for evaluating the short-term cytotoxicity of PEV@SP@EG; (B) hemolysis assay and quantitative hemolysis rates following exposure to the indicated PEV@SP@EG concentrations, with PBS and H_2_O as negative and positive controls, respectively; (C) Calcein-AM/PI staining and cell-viability quantification in macrophages treated with the indicated PEV@SP@EG concentrations; scale bar = 50 μm; (D) Annexin V-FITC/PI flow cytometry and quantification of apoptosis rates; (E) Fluorescence imaging of macrophages treated with PEV@SP@EG for 24 h. PEV was labeled with green fluorescence, EGCG with red fluorescence (TAMRA), and nuclei were counterstained with blue fluorescence (DAPI); scale bar = 25 μm. Quantitative data in panels B-D were obtained from three independent biological experiments (*n* = 3 per group), with each plotted value representing one independent experiment. ns indicates *p* ≥ 0.05.

Fig S6 *In Vivo* Safety Assessment of the PEV@SP@EG Nanosystem. Note: (A) Schematic illustration of the biosafety evaluation protocol for the PEV@SP@EG nanosystem in healthy mice; (B) H&E staining of major organs (heart, liver, spleen, lung, and kidney) to assess tissue morphology; scale bar = 50 μm; (C) Serum biochemistry analysis of hepatic function markers—ALT and AST—and renal function indicators—BUN and CRE; (D) Complete blood count analysis, including RBC, WBC, NEU, and HGB levels; (E) ELISA quantification of serum inflammatory cytokines IL-6, IFN-γ, and TNF-α. Each group included six animals. ns indicates no statistically significant difference between groups.

Fig S7 *In Vivo* Biodistribution and Pulmonary Targeting Evaluation of the PEV@SP@EG Nanosystem. Note: (A) Schematic illustration of the experimental protocol for evaluating the biodistribution and lung-targeting capability of PEV@SP@EG following intravenous administration in mice; (B) *In vivo* fluorescence imaging (IVIS Spectrum, PerkinElmer, USA) showing the whole-body distribution of SP@EG and PEV@SP@EG at various time points; (C) *Ex vivo* fluorescence imaging of major organs (heart, liver, spleen, lung, and kidney) to assess nanoparticle accumulation, including the TRALI model mice injected with PEV@SP@EG; (D) Quantitative analysis of fluorescence intensity in lung tissues across the PBS, SP@EG, and PEV@SP@EG formulation groups; (E) CLSM (Leica Microsystems, Germany) visualization of PEV@SP@EG distribution within lung tissue. PEV is labeled in red (DiR), and cell nuclei are counterstained with DAPI (blue); scale bar = 100 μm. Each group included six animals. ****p* < 0.001, *****p* < 0.0001 compared between groups.

Fig S8 Statistical Sensitivity and Power Analysis for the Quantitative Readouts in Fig. 9. Note: One-way ANOVA sensitivity curve for four groups with six biologically independent mice per group (total *N* = 24) at *α* = 0.05. The detectable omnibus effect-size thresholds were approximately Cohen's *f* = 0.744 at 80% power and *f* = 0.849 at 90% power. Endpoint-specific observed *η*^*2*^, Cohen's *f*, and retrospective power estimates for the nine quantitative readouts in Fig. 9 are provided in Table S6.

Fig S9 Design-Specific Statistical Sensitivity of Cell-Based Quantitative Measurements Based on Three Independent Biological Experiments per Group. Note: (A) Power as a function of Cohen's *d* for a two-tailed independent-samples *t*-test with three independent biological experiments per group at *α* = 0.05. Detectable thresholds were *d* = 3.071 at 80% power and *d* = 3.589 at 90% power. (B) Power as a function of Cohen's *f* for fixed-effects omnibus one-way ANOVA designs with three independent biological experiments per group at *α* = 0.05. For four-, five-, and six-group designs, the 80% power thresholds were *f* = 1.223, 1.126, and 1.053, respectively, and the 90% thresholds were *f* = 1.403, 1.285, and 1.198, respectively. Measurement-specific results are provided in Table S7.
