# Supplementary material for: A ROS-responsive platelet extracellular vesicle biomimetic nanosystem regulates CD36/Fyn-CXCL-NETosis pathway-mediated immune-inflammatory amplification in the treatment of transfusion-related acute lung injury
Source: Redox Biol. 2026 Sep 6;97:104375. doi: 10.1016/j.redox.2026.104375 (PMC13580058; doi:10.1016/j.redox.2026.104375)
Supplement: Supplementary file 1 [file mmc10.pdf]

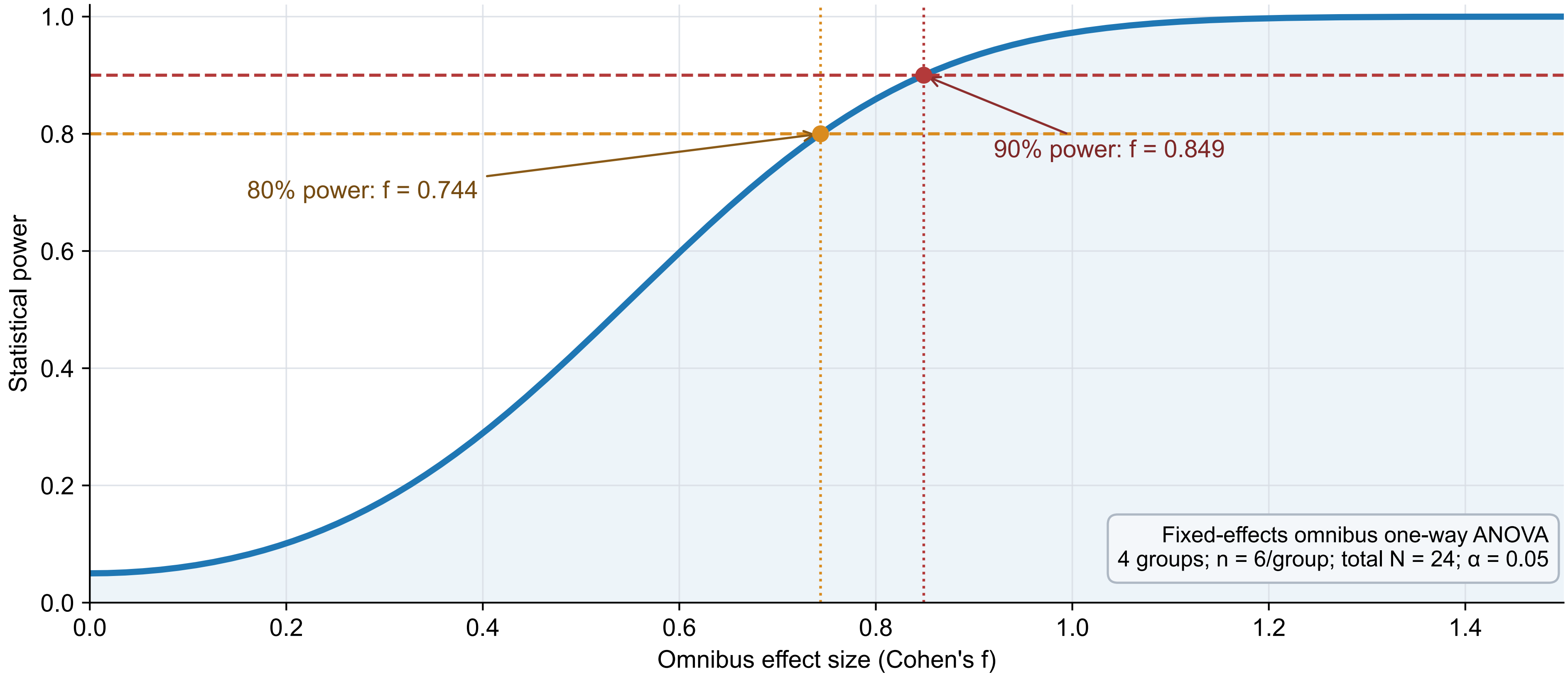
1.0
0.8
0.6
0.4
0.2
0.0
Statistical power
0.0
0.2
0.4
0.6
0.8
1.0
1.2
1.4
Omnibus effect size (Cohen's f)
80% power: f = 0.744
90% power: f = 0.849
Fixed-effects omnibus one-way ANOVA
4 groups; n = 6/group; total N = 24; α = 0.05
