# Supplementary material for: A ROS-responsive platelet extracellular vesicle biomimetic nanosystem regulates CD36/Fyn-CXCL-NETosis pathway-mediated immune-inflammatory amplification in the treatment of transfusion-related acute lung injury
Source: Redox Biol. 2026 Sep 6;97:104375. doi: 10.1016/j.redox.2026.104375 (PMC13580058; doi:10.1016/j.redox.2026.104375)
Supplement: Supplementary file 2 [file mmc11.pdf]

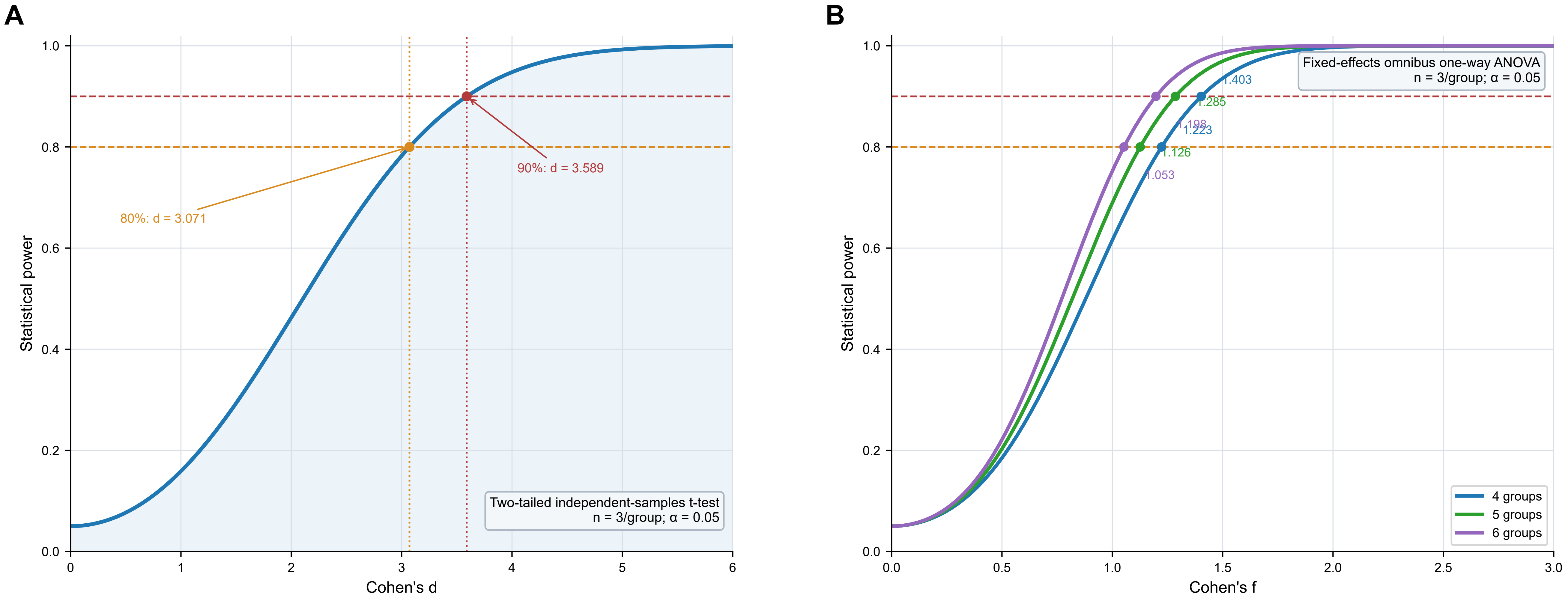

A
Statistical power
Cohen's d
80%: d = 3.071
90%: d = 3.589
Two-tailed independent-samples t-test
n = 3/group; α = 0.05
0.0
0.2
0.4
0.6
0.8
1.0
0
1
2
3
4
5
6
B
Statistical power
Cohen's f
Fixed-effects omnibus one-way ANOVA
n = 3/group; α = 0.05
1.403
1.285
1.198
1.223
1.126
1.053
4 groups
5 groups
6 groups
0.0
0.5
1.0
1.5
2.0
2.5
3.0
