# Supplementary material for: A ROS-responsive platelet extracellular vesicle biomimetic nanosystem regulates CD36/Fyn-CXCL-NETosis pathway-mediated immune-inflammatory amplification in the treatment of transfusion-related acute lung injury
Source: Redox Biol. 2026 Sep 6;97:104375. doi: 10.1016/j.redox.2026.104375 (PMC13580058; doi:10.1016/j.redox.2026.104375)
Supplement: Supplementary file 3 [file mmc1.docx]

**Table S1. Dose selection of PEV@SP@EG.**

| **No.** | Control | 5 mg/kg | 10 mg/kg | 20 mg/kg |
| --- | --- | --- | --- | --- |
| PaO₂ | 100 | 91 | 70^*^ | 65^*^ |
| PaO₂/FiO₂ | 500 | 469 | 400^*^ | 370^*^ |

Table S2. BALF total and differential cell count.

|  | Total cell count (mean ± SD) | % PMN | % Macrophage |
| --- | --- | --- | --- |
| PBS | 8.2±0.4×10^4^ | 97.2 | 1.7 |
| SP@EG | 8.2±0.4×10^4^ | 98.1 | 1.5 |
| PEV@SP@EG | 8.2±0.4×10^4^ | 97.1 | 1.8 |
| TRALI | 9.1±1.2×10^4*^ | 98.9^*^ | 1.0^*^ |
| TRALI+PEV@SP@EG | 8.7±0.7×10^4*^ | 97.2^*^ | 1.5^*^ |

**Table S3. The biological distribution of PEV@SP@EG in the major organs of mice.**

| **No.** | Organs | Distribution(%) |
| --- | --- | --- |
| 1 | Heart | 17.24 |
| 2 | Liver | 16.39 |
| 3 | Spleen | 15.58 |
| 4 | Lung | 29.03 |
| 5 | Kidney | 21.76 |

**Table S4. Sequences of primers used for RT-PCR.**

| **Gene** | **Primer Type** | **Sequence(5'->3')** |
| --- | --- | --- |
| iNOS | Forward | GCCCAGCCAGCCCAAC |
|  | Reverse | GCACATCAAAGCGGCCATAG |
| CD86 | Forward | CTTACGGAAGCACCCACGAT |
|  | Reverse | TGTAAATGGGCACGGCAGAT |
| ARG1 | Forward | ACATTGGCTTGCGAGACGTA |
|  | Reverse | ATCACCTTGCCAATCCCCAG |
| CD206 | Forward | GTCAGAACAGACTGCGTGGA |
|  | Reverse | AGGGATCGCCTGTTTTCCAG |
| GADPH | Forward | CTTCTCCTGCAGCCTCGT |
|  | Reverse | ACTGTGCCGTTGAATTTGCC |

**Table S5. Lentiviral transfection primer sequence.**

| **Name** | **Primer Sequence (5'-3')** |
| --- | --- |
| sh-CD36-1 | CCAGGGTAAGACACAGTGATA |
| sh-CD36-2 | GCCATAATTGAGTCCTATAAA |
| sh-CD36-3 | CGGATCTGAAATCGACCTTAA |
| sh-Fyn-1 | CCTGTATGGAAGGTTCACAAT |
| sh-Fyn-2 | GCTCTGAAGTTGCCAAACCTT |
| sh-Fyn-3 | CATCCCGAACTACAACAACTT |
| sh-NC | TTCTCCGAACGTGTCACGT |
