# Supplementary material for: A ROS-responsive platelet extracellular vesicle biomimetic nanosystem regulates CD36/Fyn-CXCL-NETosis pathway-mediated immune-inflammatory amplification in the treatment of transfusion-related acute lung injury
Source: Redox Biol. 2026 Sep 6;97:104375. doi: 10.1016/j.redox.2026.104375 (PMC13580058; doi:10.1016/j.redox.2026.104375)
Supplement: Supplementary file 4 [file mmc3.zip › redox_104375_mmc3.pdf]

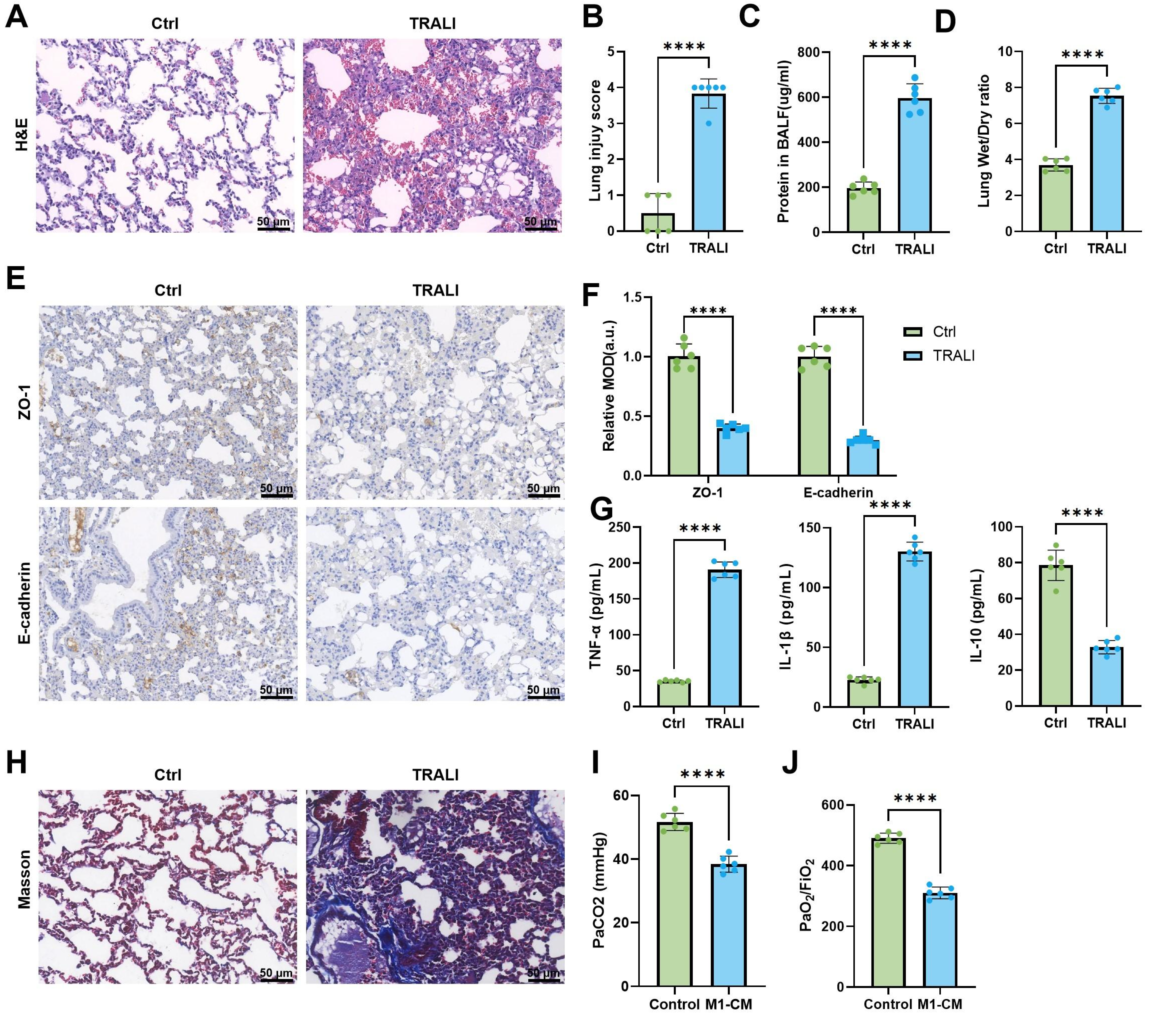
A
Ctrl
TRALI
H&E
50 μm
B
Lung injuy score
****
Ctrl
TRALI
C
Protein in BALF(ug/ml)
****
D
Lung Wet/Dry ratio
****
E
ZO-1
E-cadherin
F
Relative MOD(a.u.)
ZO-1
E-cadherin
Ctrl
TRALI
G
TNF-α (pg/mL)
IL-1β (pg/mL)
IL-10 (pg/mL)
H
Masson
I
PaCO2 (mmHg)
Control
M1-CM
J
PaO2/FiO2
