# Supplementary material for: A ROS-responsive platelet extracellular vesicle biomimetic nanosystem regulates CD36/Fyn-CXCL-NETosis pathway-mediated immune-inflammatory amplification in the treatment of transfusion-related acute lung injury
Source: Redox Biol. 2026 Sep 6;97:104375. doi: 10.1016/j.redox.2026.104375 (PMC13580058; doi:10.1016/j.redox.2026.104375)
Supplement: Supplementary file 5 [file mmc4.zip › redox_104375_mmc4.pdf]

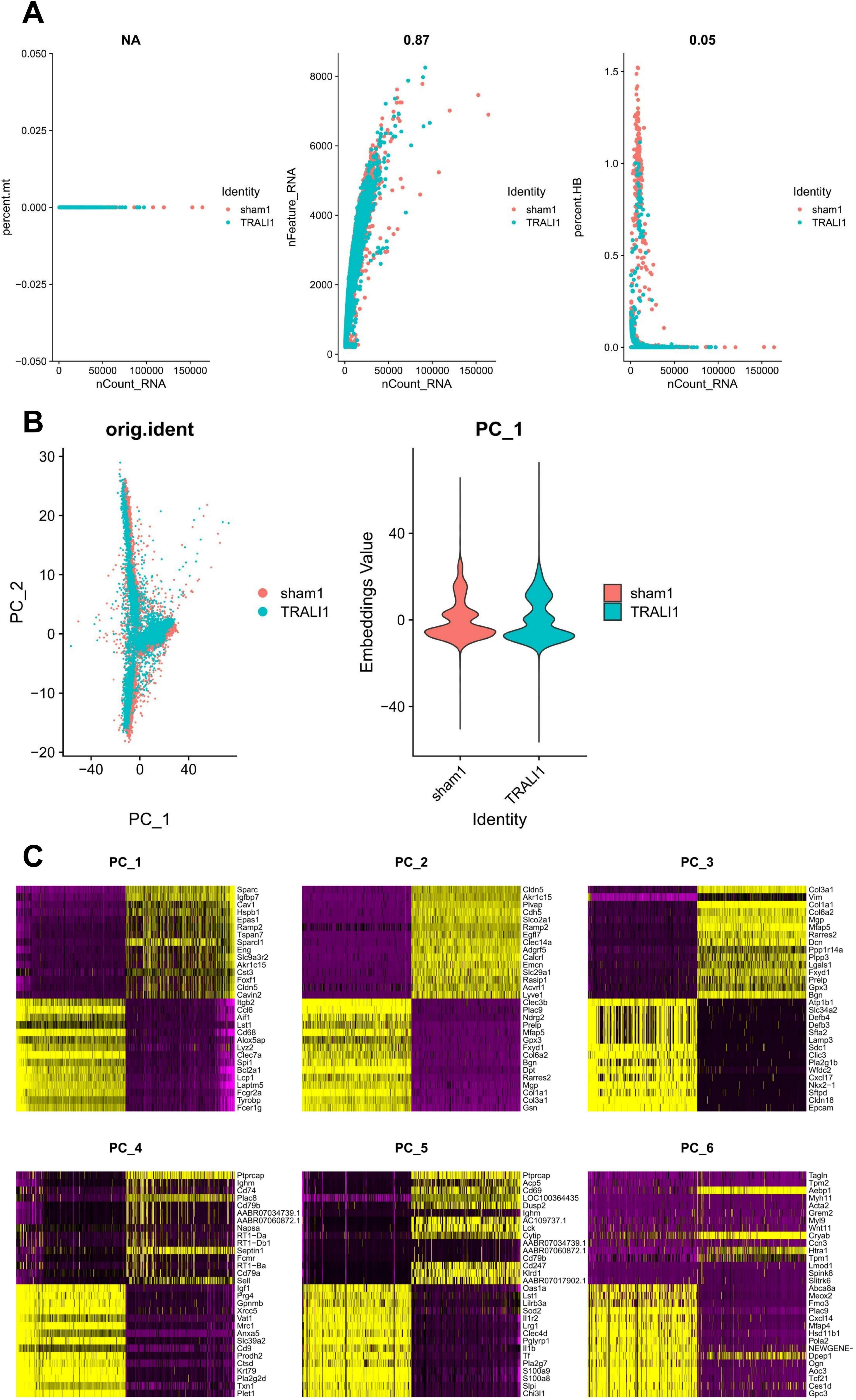

A
NA
0.87
0.05
percent.mt
nFeature_RNA
percent.HB
nCount_RNA
Identity
sham1
TRALI1
B
orig.ident
PC_1
PC_2
Embeddings Value
Identity
C
PC_1
Sparc
Igfbp7
Cav1
Hspb1
Epas1
Ramp2
Tspan7
Sparcl1
Eng
Slc9a3r2
Akr1c15
Cst3
Foxf1
Cldn5
Cavin2
Itgb2
Ccl6
Aif1
Lst1
Cd68
Alox5ap
Lyz2
Clec7a
Spi1
Bcl2a1
Lcp1
Laptm5
Fcgr2a
Tyrobp
Fcer1g
PC_2
Cldn5
Akr1c15
Plvap
Cdh5
Slco2a1
Ramp2
Egfl7
Clec14a
Adgrf5
Calcrl
Emcn
Slc29a1
Rasip1
Acvrl1
Lyve1
Clec3b
Plac9
Ndrg2
Prelp
Mfap5
Gpx3
Fxyd1
Col6a2
Bgn
Dpt
Rarres2
Mgp
Col1a1
Col3a1
Gsn
PC_3
Col3a1
Vim
Col1a1
Col6a2
Mgp
Mfap5
Rarres2
Dcn
Ppp1r14a
Plpp3
Lgals1
Fxyd1
Prelp
Gpx3
Bgn
Atp1b1
Slc34a2
Defb4
Defb3
Sfta2
Lamp3
Sdc1
Clic3
Pla2g1b
Wfdc2
Cxcl17
Nkx2−1
Sftpd
Cldn18
Epcam
PC_4
Ptprcap
Ighm
Cd74
Plac8
Cd79b
AABR07034739.1
AABR07060872.1
Napsa
RT1−Da
RT1−Db1
Septin1
Fcmr
RT1−Ba
Cd79a
Sell
Igf1
Prg4
Gpnmb
Xrcc5
Vat1
Mrc1
Anxa5
Slc39a2
Cd9
Prodh2
Ctsd
Krt79
Pla2g2d
Txn1
Plet1
PC_5
Ptprcap
Acp5
Cd69
LOC100364435
Dusp2
Ighm
AC109737.1
Lck
Cytip
AABR07034739.1
AABR07060872.1
Cd79b
Cd247
Klrd1
AABR07017902.1
Oas1a
Lst1
Lilrb3a
Sod2
Il1r2
Lrg1
Clec4d
Pglyrp1
Il1b
Tf
Pla2g7
S100a9
S100a8
Slpi
Chi3l1
PC_6
Tagln
Tpm2
Aebp1
Myh11
Acta2
Grem2
Myl9
Wnt11
Cryab
Ccn3
Htra1
Tpm1
Lmod1
Spink8
Slitrk6
Abca8a
Meox2
Fmo3
Plac9
Cxcl14
Mfap4
Hsd11b1
Pola2
NEWGENE-
Dpep1
Ogn
Aoc3
Tcf21
Ces1d
Gpc3
