# Supplementary material for: A ROS-responsive platelet extracellular vesicle biomimetic nanosystem regulates CD36/Fyn-CXCL-NETosis pathway-mediated immune-inflammatory amplification in the treatment of transfusion-related acute lung injury
Source: Redox Biol. 2026 Sep 6;97:104375. doi: 10.1016/j.redox.2026.104375 (PMC13580058; doi:10.1016/j.redox.2026.104375)
Supplement: Supplementary file 6 [file mmc5.zip › redox_104375_mmc5.pdf]

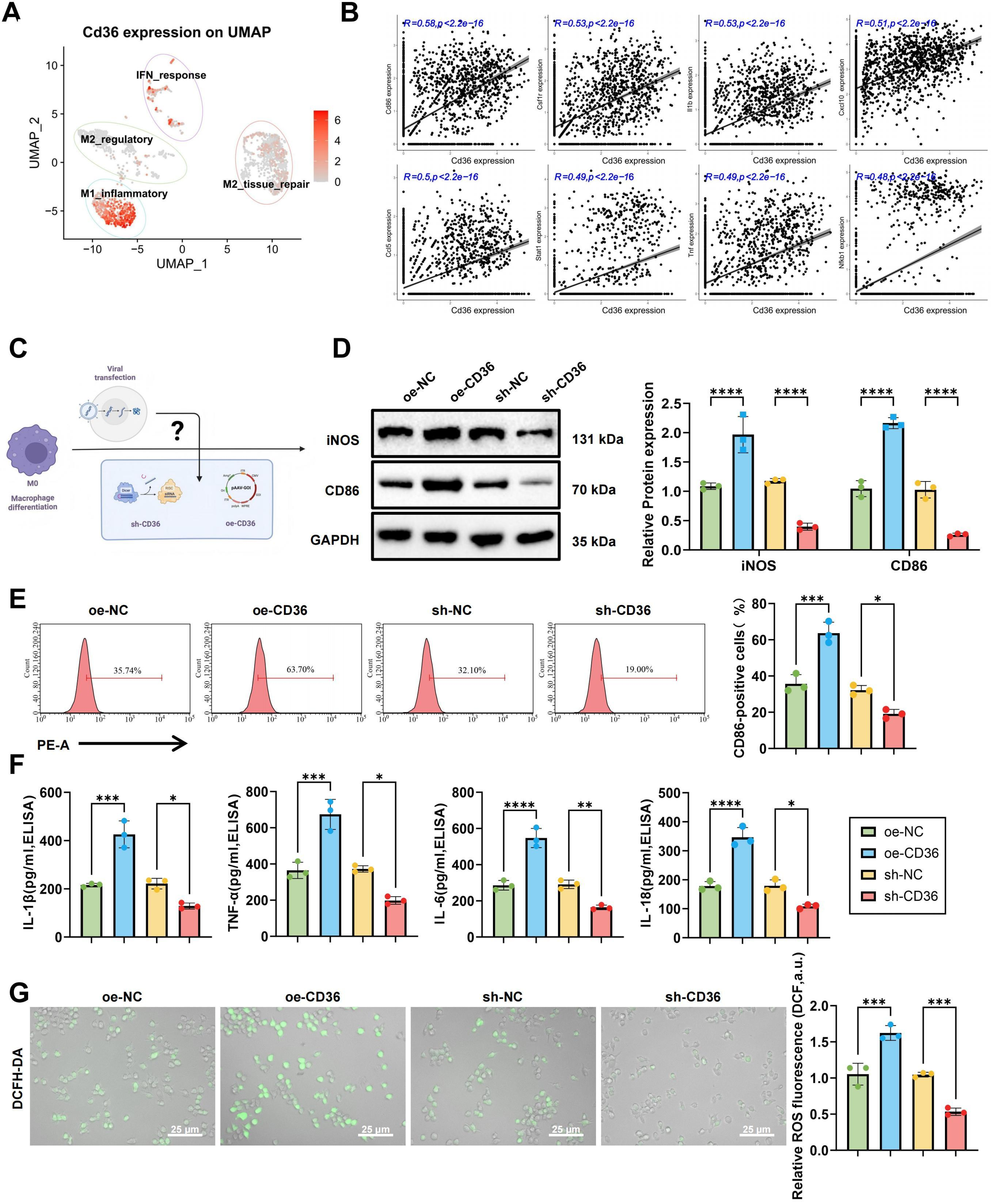
A
Cd36 expression on UMAP
IFN_response
M2_regulatory
M1_inflammatory
M2_tissue_repair
UMAP_1
UMAP_2
B
R=0.58,p<2.2e−16
R=0.53,p<2.2e−16
R=0.53,p<2.2e−16
R=0.51,p<2.2e−16
R=0.5,p<2.2e−16
R=0.49,p<2.2e−16
R=0.49,p<2.2e−16
R=0.48,p<2.2e−16
Cd36 expression
C
Viral transfection
M0
Macrophage differentiation
sh-CD36
oe-CD36
D
oe-NC
oe-CD36
sh-NC
sh-CD36
iNOS
CD86
GAPDH
131 kDa
70 kDa
35 kDa
Relative Protein expression
E
PE-A
35.74%
63.70%
32.10%
19.00%
CD86-positive cells（%）
F
IL-1β(pg/ml,ELISA)
TNF-α(pg/ml,ELISA)
IL-6(pg/ml,ELISA)
IL-18(pg/ml,ELISA)
G
DCFH-DA
25 μm
Relative ROS fluorescence (DCF,a.u.)
