# Supplementary material for: A ROS-responsive platelet extracellular vesicle biomimetic nanosystem regulates CD36/Fyn-CXCL-NETosis pathway-mediated immune-inflammatory amplification in the treatment of transfusion-related acute lung injury
Source: Redox Biol. 2026 Sep 6;97:104375. doi: 10.1016/j.redox.2026.104375 (PMC13580058; doi:10.1016/j.redox.2026.104375)
Supplement: Supplementary file 7 [file mmc6.zip › redox_104375_mmc6.pdf]

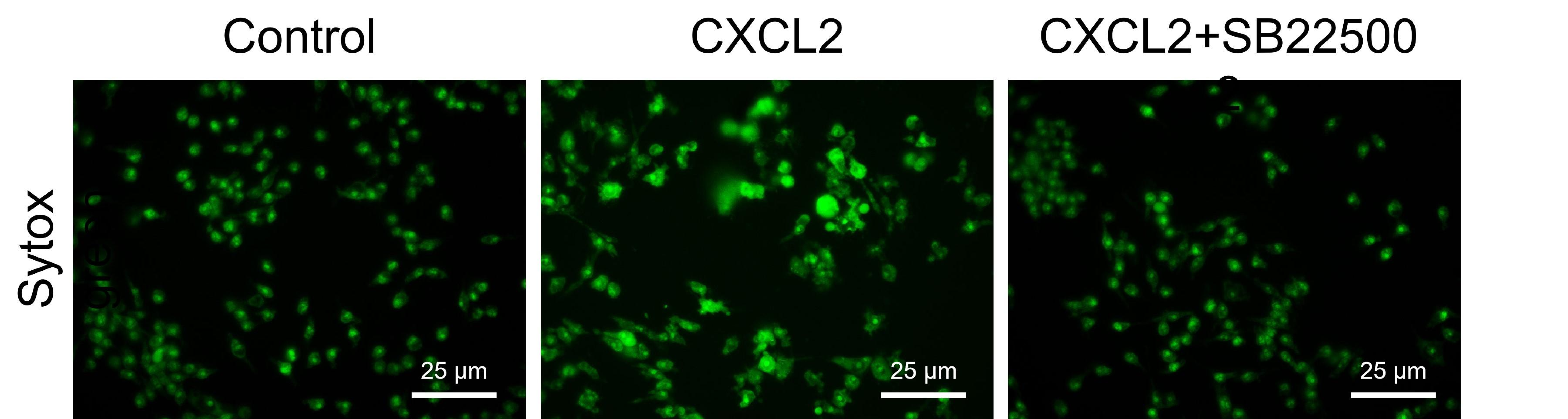
Control
CXCL2
CXCL2+SB22500
2
Sytox
25 μm
25 μm
25 μm
