# Supplementary material for: A ROS-responsive platelet extracellular vesicle biomimetic nanosystem regulates CD36/Fyn-CXCL-NETosis pathway-mediated immune-inflammatory amplification in the treatment of transfusion-related acute lung injury
Source: Redox Biol. 2026 Sep 6;97:104375. doi: 10.1016/j.redox.2026.104375 (PMC13580058; doi:10.1016/j.redox.2026.104375)
Supplement: Supplementary file 8 [file mmc7.zip › redox_104375_mmc7.pdf]

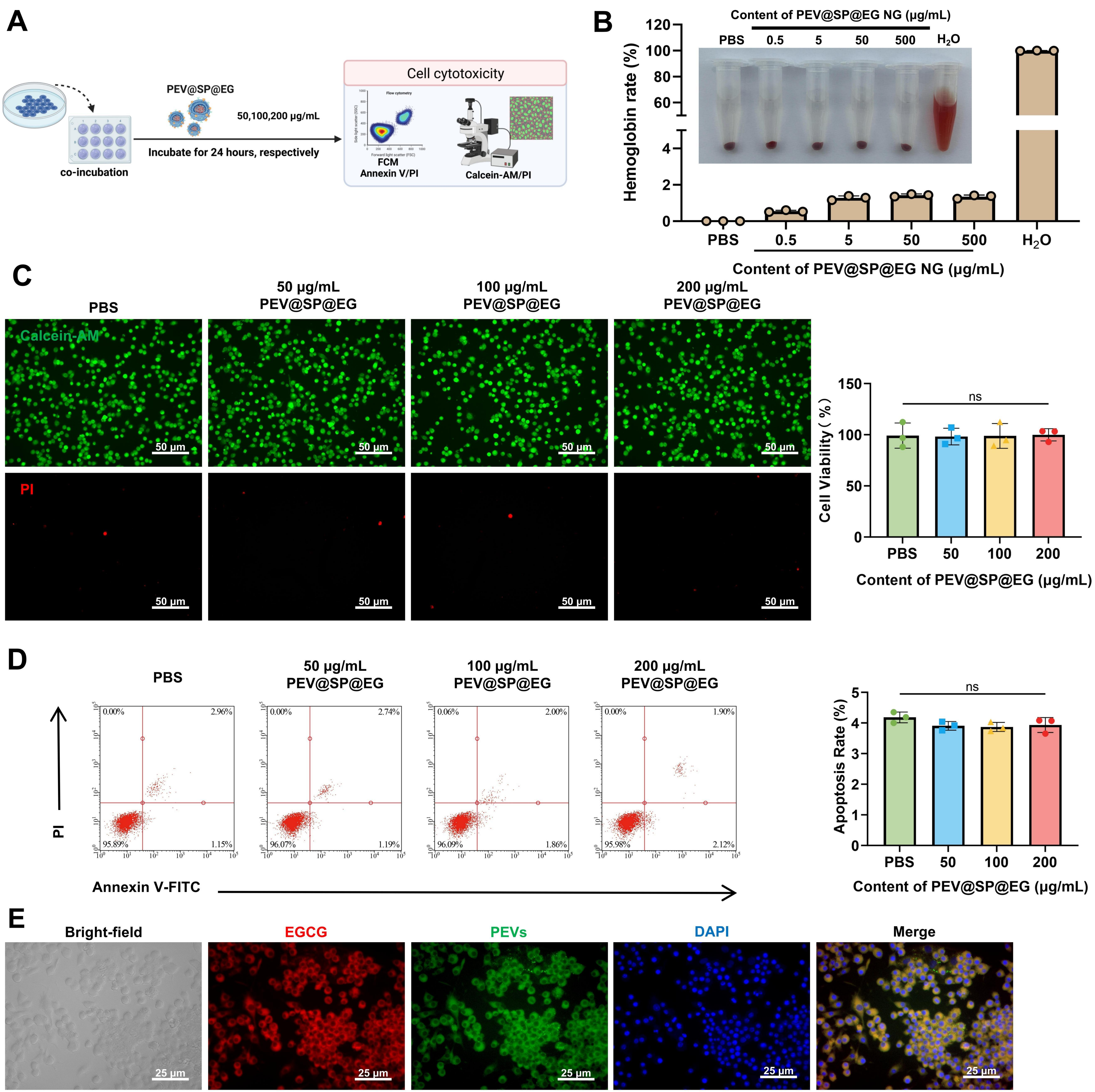

A
PEV@SP@EG
50,100,200 μg/mL
co-incubation
Incubate for 24 hours, respectively
Cell cytotoxicity
Flow cytometry
FCM
Annexin V/PI
Calcein-AM/PI
B
Content of PEV@SP@EG NG (μg/mL)
PBS
0.5
5
50
500
H2O
Hemoglobin rate (%)
C
PBS
50 μg/mL PEV@SP@EG
100 μg/mL PEV@SP@EG
200 μg/mL PEV@SP@EG
Calcein-AM
PI
50 μm
Cell Viability (%)
ns
Content of PEV@SP@EG (μg/mL)
D
0.00%
2.96%
95.89%
1.15%
2.74%
96.07%
1.19%
0.06%
2.00%
96.09%
1.86%
1.90%
95.98%
2.12%
PI
Annexin V-FITC
Apoptosis Rate (%)
E
Bright-field
EGCG
PEVs
DAPI
Merge
25 μm
