# Supplementary material for: A ROS-responsive platelet extracellular vesicle biomimetic nanosystem regulates CD36/Fyn-CXCL-NETosis pathway-mediated immune-inflammatory amplification in the treatment of transfusion-related acute lung injury
Source: Redox Biol. 2026 Sep 6;97:104375. doi: 10.1016/j.redox.2026.104375 (PMC13580058; doi:10.1016/j.redox.2026.104375)
Supplement: Supplementary file 9 [file mmc8.zip › redox_104375_mmc8.pdf]

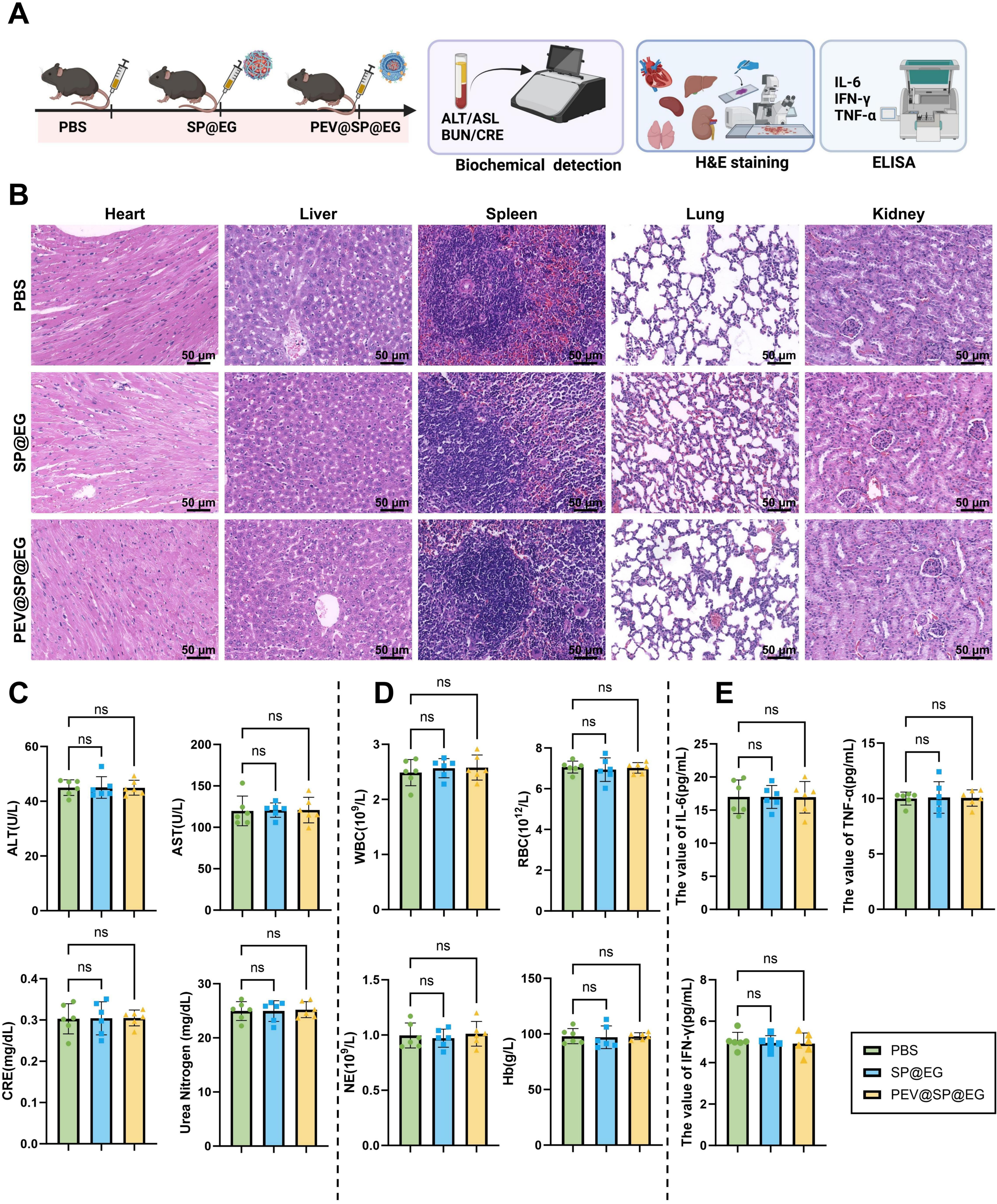

A
PBS
SP@EG
PEV@SP@EG
ALT/ASL
BUN/CRE
Biochemical detection
H&E staining
IL-6
IFN-γ
TNF-α
ELISA
B
Heart
Liver
Spleen
Lung
Kidney
PBS
SP@EG
PEV@SP@EG
50 μm
C
ALT(U/L)
AST(U/L)
CRE(mg/dL)
Urea Nitrogen (mg/dL)
D
WBC(10^9/L)
RBC(10^12/L)
NE(10^9/L)
Hb(g/L)
E
The value of IL-6(pg/mL)
The value of TNF-α(pg/mL)
The value of IFN-γ(pg/mL)
ns
PBS
SP@EG
PEV@SP@EG
