# Supplementary material for: A ROS-responsive platelet extracellular vesicle biomimetic nanosystem regulates CD36/Fyn-CXCL-NETosis pathway-mediated immune-inflammatory amplification in the treatment of transfusion-related acute lung injury
Source: Redox Biol. 2026 Sep 6;97:104375. doi: 10.1016/j.redox.2026.104375 (PMC13580058; doi:10.1016/j.redox.2026.104375)
Supplement: Supplementary file 10 [file mmc9.zip › redox_104375_mmc9.pdf]

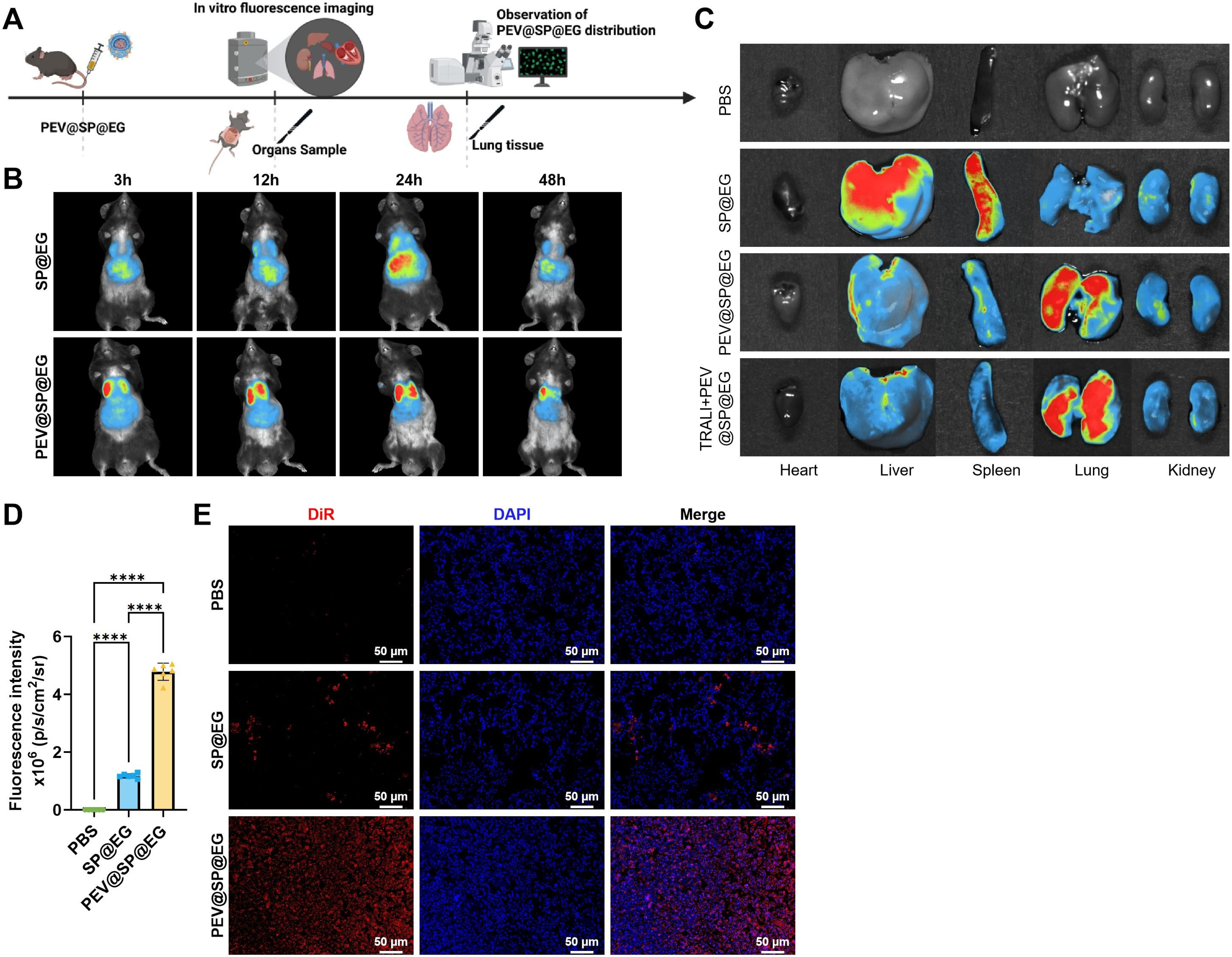
A
In vitro fluorescence imaging
Observation of
PEV@SP@EG distribution
PEV@SP@EG
Organs Sample
Lung tissue
B
3h
12h
24h
48h
SP@EG
PEV@SP@EG
C
PBS
SP@EG
PEV@SP@EG
TRALI+PEV
@SP@EG
Heart
Liver
Spleen
Lung
Kidney
D
Fluorescence intensity
x10^6 (p/s/cm^2/sr)
****
****
****
0
2
4
6
PBS
SP@EG
PEV@SP@EG
E
DiR
DAPI
Merge
PBS
SP@EG
PEV@SP@EG
50 μm
